# Advancements in tailored polymeric membranes for microbial fuel cells: a comprehensive review of recent developments and challenges

**DOI:** 10.1039/d5ra01149c

**Published:** 2025-05-13

**Authors:** Elangovan Mahendiravarman, Natarajan Rajamohan, Manivasagan Rajasimman, Sankar Sudharsan Rameshwar, Iyman Abrar

**Affiliations:** a Department of Chemical Engineering, Annamalai University Annamalainagar 608 002 Tamilnadu India mahendiravarman@gmail.com; b Chemical Engineering Section, Faculty of Engineering, Sohar University Sohar PC-311 Oman rnatarajan@su.edu.om; c Department of Chemical Engineering, Birla Institute of Technology and Science, Pilani Hyderabad Campus, Jawahar Nagar, Kapra Mandal, Medchal District Telangana 500078 India iyman@hyderabad.bits-pilani.ac.in

## Abstract

The global bioenergy research community is very interested in the microbial fuel cell (MFC), a biofuel conversion technology that cleanses wastewater and produces power at the same time. Separators have come a long way, but problems like oxygen leakage and limited proton transfer still exist. These issues cause internal resistance and lower MFC performance, which restricts the practical use of separators. This review provides a thorough analysis of the latest membrane separators that are appropriate for MFCs, explaining their components, operating principles, and major performance-affecting elements such pH splitting problems, oxygen and substrate crossover, membrane resistance, and biofouling. Various membrane materials are explored, such as porous materials like textiles, glass fibers, and polymer, microfiltration and ultrafiltration membranes, and ion exchange membranes (anion, cation, and bipolar). Specifically, characteristic ionic groups that are essential for the best MFC performance are what make anion exchange membranes (AEMs) and cation exchange membranes (CEMs) stand out. In addition, it provides a thorough overview of customized polymeric membranes for MFCs, including their function, necessary characteristics, advantages, types, structures, uses, manufacturing processes, characterization techniques, and strategies to enhance performance. This study emphasizes the crucial role of tailored polymeric membranes in advancing MFC technology for sustainable energy generation, while also exploring their future potential for enhanced performance.

## Abstract 2

The global bioenergy research community is very interested in the microbial fuel cell (MFC), a biofuel conversion technology that cleanses wastewater and produces power at the same time.

## Graphical abstract

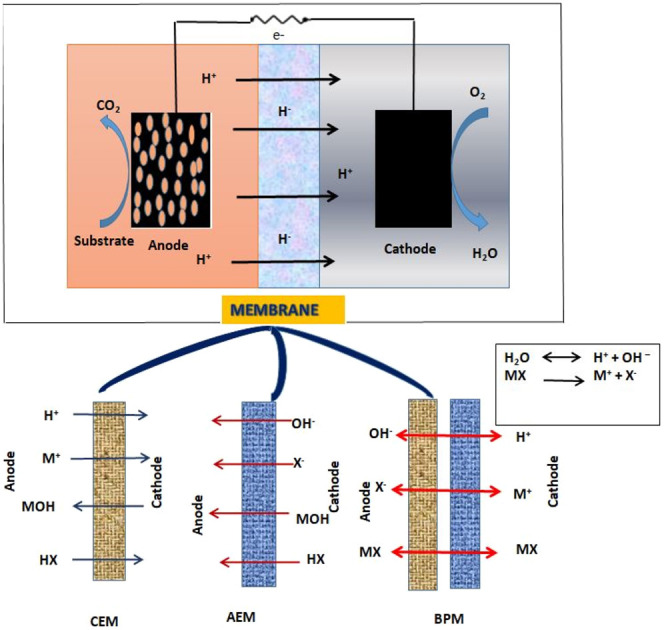

## 1. Introduction

The rapid utilization of energy and fast depletion of fossil fuel sources have created unforeseen pressure on the energy sector. With the global climate action plan and the predicted average global temperature rise of about 3.6 °C, new directions on energy generation with renewable sources have gained momentum. The utilization of renewable energy has grown from 2% to 6.7% in the past decade, up to 2021.^1^ A distinction between renewable and non-renewable energy sources is made for all sources of energy on earth.^2^ The promise of renewable energy sources and others has not yet been realized. Instead, non-renewable energy sources like fossil fuels, which really account for most energy usage, have already reached the point of depletion. Fossil fuels seriously damage the environment by emitting greenhouse gases such CO_2_, CO, CH_4_, NO and SO_2_ among others. Due to factors like acid rain, climate change, and global warming, the use of fossil fuels has seriously endangered not only human existence but also that of flora and fauna.^3^ Nuclear energy, another non-renewable energy source, is advancing despite numerous environmental issues like radiation risks, *etc.*^4^ This deadlock has forced nations to explore renewable energy technologies like solar and wind. However, achieving scalability and long-term sustainability remains a significant challenge. In this regard, MFCs have gained attention as an innovative bio-electrochemical system that addresses two critical issues: clean energy production and environmental cleanup.^5^ In 1911, Potter reports the MFC. By utilizing the metabolic processes of microorganisms, MFCs can treat wastewater while generating electricity. This combination of energy generation and waste treatment highlights the potential of MFCs in advancing renewable energy technologies.

The efficiency of MFCs largely depends on advanced membrane technologies that create a barrier between the anode and cathode chambers. These membranes facilitate selective ion transfer while preventing the mixing of fuel and oxidants.^6^ Polymeric membranes engineered for properties such as robustness, ion selectivity, proton conductivity, and resistance to fouling have demonstrated considerable promise in enhancing MFC performance.^6,7^ Building on these advancements, considerable progress has been achieved in the development, manufacturing processes, testing procedures, and uses of customized polymeric membranes for MFCs in the last few years. Because of the distinct design of these membranes, MFCs can operate with greater efficiency, greater power output, and a longer lifespan. Several manufacturing processes have been used to produce membranes with suitable impacts and properties, including as layer-by-layer assembly, electrospinning, and solution casting.^8–10^ The performance of these membranes is assessed using characterization techniques such ion exchange capacity measurement, water uptake, swelling behavior, and electrochemical impedance spectroscopy.^11,12^

**Fig. 1:**
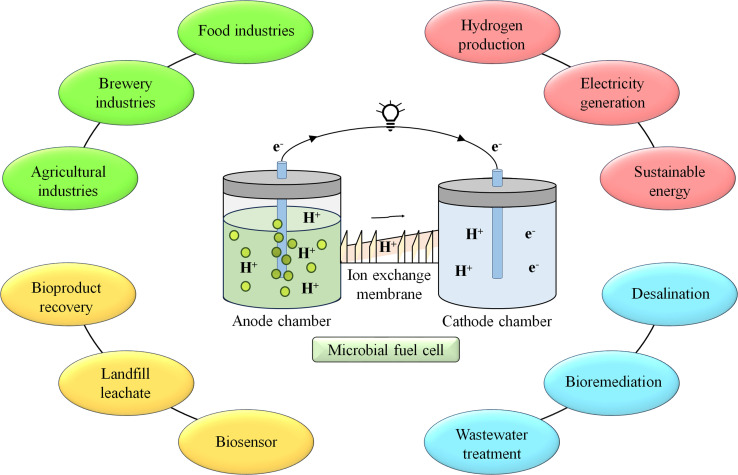
Application of MFC in various fields.

Tailored polymeric membranes have a wide range of significant uses in MFCs. To improve the effectiveness and applicability of MFC technology, these membranes are used in proton exchange, selective ion exchange, antifouling, and nanocomposite membranes, among other applications.^12^ Nonetheless, there are still several issues to be resolved, such as enhancing ion transport efficiency, robustness, affordability, scalability, and mitigation of biofouling. It will be imperative to tackle these obstacles and investigate novel avenues in membrane science to propel the area of MFCs toward environmentally friendly energy production and remediation.^12^ The MFC finds application in many fields like wastewater treatment land fill leachate, energy recovery^9^ and it is depicted in Fig. 1.

**Fig. 2:**
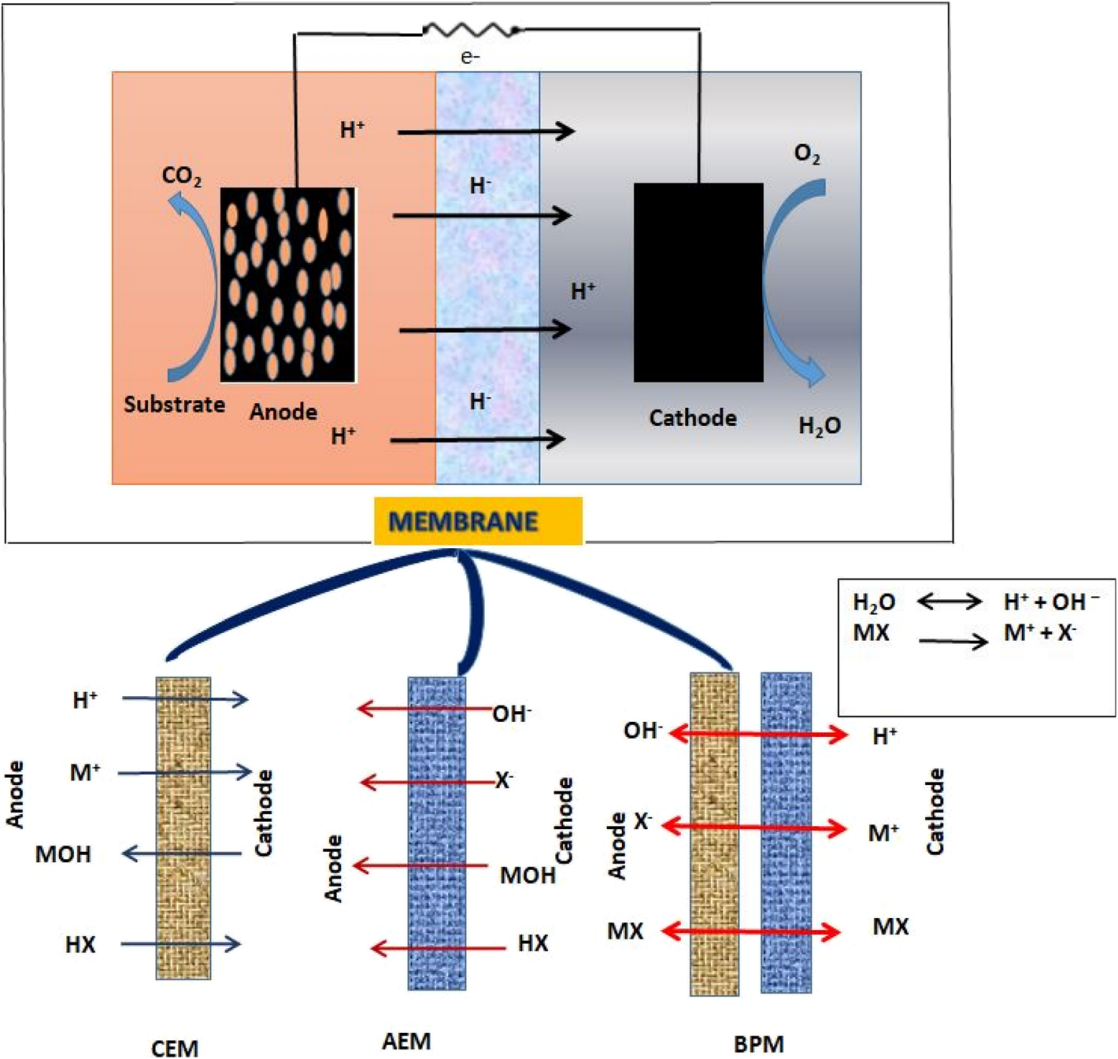
Dual chambered MFC system with anode, cathode and membrane.

Fig. 2 illustrates how an MFC generally comprises of anode and cathode compartments that are physically divided by a PEM.^7^ The substrates which are organic in nature undergoes oxidative changes to emit both negatively charged electrons and positively charged protons. Due to cathodic reaction, oxygen is reduced to water and proton and electron interactions occur.^12^ The active biocatalyst oxidizes the carbon sources or substrates in the anode compartment to generate electrons and protons. The anodic reaction of glucose is used as an example in eqn (1.1). The production of electricity may be hampered by the presence of oxygen in the anode chamber. To maintain a practical system, the bacteria must be kept as far away from the oxygen source as feasible. It is simple to separate the biocatalyst from oxygen that only allows charge passage between the electrodes.^13^

1.1 C_6_H_12_O_6_ + 6H_2_O + 6O_2_ → 6CO_2_ + 12H_2_O

1.2 C_6_H_12_O_6_ + 6H_2_O → 6CO_2_ + 24H^+^ + 24e^−^

MFCs can be divided into two categories based on how actively growing microorganisms transfer electrons from media to an anode electrode: MFCs with mediator and mediator less MFCs.^14^ Despite great results in the lab, the MFC technique faced numerous difficulties when it came to scaling up and practical implementation, including compartment turbulence and membrane resistance during the process of transporting. The internal resistance created between the chambers, is a factor deciding much power is generated in MFCs based on substrate concentration, and the output is lowered.^15^ Absence of PEM in MFCs such single-chamber MFCs (SCMFC), stacked MFCs, and up flow MFCs is observed to lower internal resistance, which leads to improved output.^16^

It was known that wastes from industries, humans, and animals, which are rich in organic compounds, might be utilized to produce power. For instance, it has been discovered that adding urine to MFCs as a carbon and nitrogen source helps the voltage-producing microbes produce more electricity. The two benefits of wastewater purification and electricity production are the most intriguing. The wastewater, which has been cleaned off by 80%, can be used for gardens, quenching plants for companies, and cooling towers. The overall performance of MFCs as assessed by power density, cell voltage, *etc.* is greatly influenced by the consumption of oxygen in the cathode chamber, the oxidation of substrates in the anode chamber, the movement of electrons between the anode compartment and the anode surface, and the permeability of the proton exchange membrane.^16^

Usually, dead bacteria, plankton, fecal matter, and anthropogenic elements can be found in soil sediments that have plant and animal debris.^2^ Sediments are a useful material for power generation due to their organic content, which ranges from 0.4% to 2.2% by weight.^17^ Exo electrogene use these substances to transport electrons outside of the cell. As a result, a sediment-type MFC has a cathode suspended in the water above the anaerobic silt as its anode.^17^

An ion exchange membrane divides the anode and cathode compartments of a simple double-chambered MFC, which are connected by an external electric circuit shown in Fig. 2. A bio-anode, which is an anode substrate covered with a film of bacteria (the catalyst) and submerged in the solution of organic matter, is present in the anode compartment. It may be fed constantly or sporadically. The fuel (the electron donor) is oxidized by the bacterial metabolism, which also releases electrons and protons. The cathode compartment is reached by the electrons that were transmitted to the anode substrate through particular processes, such as direct contact, nanowires, or mediators, *etc.* A reduction reaction at the cathode compartment in the presence of oxygen produces water at the cathode. The reduction reaction could be catalysed by a biofilm. A normal anaerobic bacterium is an anodophile, whereas a cathodophile is the exact opposite. Another design does not require a cathode chamber because the cathode is immediately exposed to air, just like in single chamber MFCs. Concentration polarisation is the total of the energy losses due to mass transfer, the formation of reaction products, and the depletion of reactants in the electrolyte close to the two electrodes. The concentration gradient is lowered with the aid of stirring and/or bubbling. Ohmic losses are caused by the movement of electrons *via* the electrodes, external resistance, current collectors, contacts, and ionic flux through the electrolyte, in addition to other factors. By employing membranes with low resistance, reducing the electrode spacing, and adjusting the solution conductivity conducive to bacterial viability, ohmic losses can be minimized. An MFC's evaluation in terms of power production, current output, and efficiency is based on factors including the kind and performance of the electrode materials, system configuration, and operating circumstances, among others.

### 1.1 Timeline and evolution of membrane research in microbial fuel cells

**Fig. 3:**
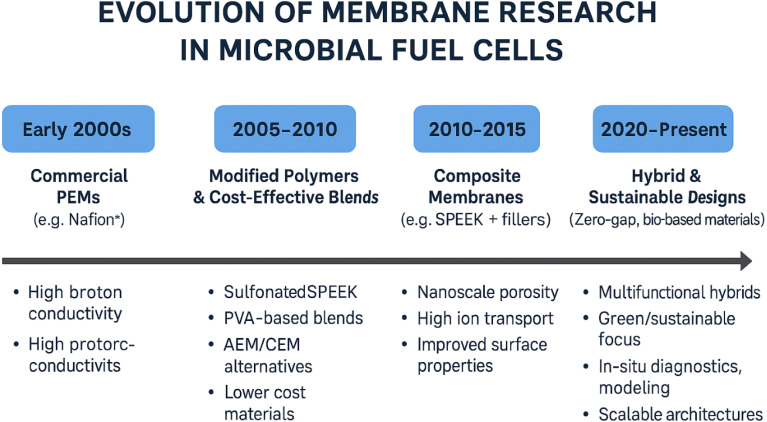
Timeline and evolution of membrane research in microbial fuel cells.

The development of membrane technologies in microbial fuel cells (MFCs) has undergone significant transformation over the past two decades, shaped by the evolving demands of system performance, cost-effectiveness, and long-term operational stability. The timeline of this progression reflects key shifts in material design, fabrication techniques, and the understanding of ion transport phenomena in BES. The timeline and evolution of membrane research in MFCs in Fig. 3.

#### 1.1.1 Early 2000s – commercial PEMs and the foundation of MFC membrane research

Initial studies on MFCs widely employed commercially available proton exchange membranes (PEMs), such as Nafion®, due to their high proton conductivity and proven use in fuel cell technologies.^15^ These membranes played a crucial role in establishing foundational knowledge regarding membrane function in bioelectrochemical systems. However, limitations such as high material cost, oxygen crossover, low chemical stability in biological environments, and poor resistance to biofouling led to an increasing interest in alternatives.^15,17^

#### 1.1.2 2005–2010 – exploration of modified polymers and cost-effective alternatives

This period marked the shift toward developing sulfonated polymers such as sulfonated polyether ether ketone (SPEEK)^18^ and polyvinyl alcohol (PVA) blends^19^ as lower-cost substitutes for PEMs. Research focused on tailoring these materials to balance ionic conductivity and mechanical integrity. Meanwhile, anion exchange membranes (AEMs) and cation exchange membranes (CEMs) were introduced for alkaline and microbial desalination cell applications, further diversifying membrane functionalities across BES platforms.^17^

#### 1.1.3 2010–2015 – emergence of composite membranes

The next major advancement came with the introduction of composite membranes, which integrated inorganic fillers (*e.g.*, TiO_2_, SiO_2_, Fe_3_O_4_) and carbon-based nanomaterials (*e.g.*, graphene oxide, MWCNTs) into polymer matrices.^21–23,29^ These composites improved not only proton and hydroxide ion conductivity, but also provided enhanced mechanical strength, oxidative stability, and resistance to biofouling. This period also witnessed a growing interest in multi-functional membranes capable of withstanding complex biochemical and electrochemical environments.

#### 1.1.4 2015–2020 – development of nanostructured membranes

Advanced fabrication techniques such as electrospinning, layer-by-layer (LbL) assembly, and template-assisted synthesis enabled the creation of nanostructured membranes with superior ion transport capabilities and surface properties.^9^ These membranes featured nanoscale porosity, high surface area, and controlled functional group distribution, leading to significant performance improvements in terms of ionic conductivity, microbial adhesion, and operational stability. The integration of tailored nanostructures represented a major step toward bridging membrane science with materials engineering for MFC applications.^9^

#### 1.1.5 2020–present – hybrid membranes and sustainable innovations

Recent trends emphasize the development of hybrid membranes, which combine surface modification, nanostructuring, and composite strategies to produce membranes with synergistic properties. Research has also shifted toward bio-based, biodegradable, and green-sourced materials, aligned with sustainability goals.^13,24–26^ Simultaneously, zero-gap configurations, membraneless designs, and scalable membrane fabrication processes are being explored to reduce system cost and complexity. Innovations in computational modeling and *in situ* diagnostic techniques now support more rational membrane design by elucidating the dynamics of ion transport, fouling, and structural degradation under operational conditions.^27^

This review paper aims to give a thorough overview of the tailored polymeric membranes used in MFCs. The article covers the function of membranes in MFCs, the characteristics necessary for MFC membranes to become efficient, and the benefits of utilizing polymeric membranes. Furthermore, included are the several tailored polymeric membranes used in MFCs, such as cation exchange membranes, anion exchange membranes, and bipolar membranes, along with details on their uses, designs, and properties. The article also discusses methods for improving the performance of these membranes as well as various manufacturing and characterization techniques. A discussion of current applications challenges, and potential uses of tailored polymeric membranes in MFCs.

## 2. Role of membranes in MFC

Membranes serve as a fundamental component in Bioelectrochemical Systems (BES), including Microbial Fuel Cells, as they act as separators between the anode and cathode compartments while selectively allowing the transport of specific ions. Their primary role is to maintain ionic conductivity while preventing the direct mixing of anolyte and catholyte solutions, which would otherwise lead to short-circuiting and reduced efficiency.^7^ The choice of membrane material significantly impacts power generation, internal resistance, substrate utilization, and the overall stability of BES. Various types of membranes, including Proton Exchange Membranes (PEMs), AEMs, Cation Exchange Membranes (CEMs), Bipolar Membranes (BPMs), and Nanocomposite Membranes, have been explored to enhance BES performance.^17^

Proton Exchange Membranes, such as Nafion and Sulfonated Polyether Ether Ketone (SPEEK), are widely used due to their excellent proton conductivity and chemical stability. These membranes facilitate the selective transport of H^+^ ions from the anode to the cathode while preventing oxygen crossover. However, despite their high efficiency, PEMs suffer from challenges such as high cost, biofouling, and proton leakage, which can cause a pH imbalance in BES. Anion Exchange Membranes, such as QA-AEM, Ralex AEM, and PVA-AEM, provide an alternative to PEMs by enabling the selective transport of negatively charged ions like OH^−^ and Cl^−^. AEMs are particularly beneficial in alkaline microbial fuel cells, as they help maintain an optimal pH environment. However, their conductivity is generally lower than that of PEMs, and they may suffer from stability issues in long-term applications.^28^

Another category is Cation Exchange Membranes, such as Zero-gap CEMs and Nafion-based CEMs, which allow the movement of cations (Na^+^, K^+^, NH_4_^+^) across the membrane. These membranes are useful for maintaining ionic balance and reducing pH gradients in microbial fuel cells.^27^ However, their performance can be affected by biofouling, scaling, and high resistance, which may reduce ion transfer efficiency. Bipolar Membranes (BPMs) offer a unique advantage by enabling both anion and cation transport while also promoting water dissociation into H^+^ and OH^−^ ions, which helps in pH regulation within BES. However, higher resistance and energy loss are some of the limitations associated with BPMs.

In addition to conventional ion-exchange membranes, nanocomposite and hybrid membranes have gained attention for their enhanced conductivity, mechanical strength, and durability. Examples include SPEEK/MMT (Montmorillonite),^29^ QPSU/GO (Graphene Oxide),^30^ and Zr-MOF/PVDF (Metal–Organic Frameworks with Polyvinylidene Fluoride),^31^ which integrate inorganic nanoparticles or functionalized polymers to improve electrochemical properties. These membranes enhance ion selectivity, water uptake, and thermal stability, making them promising candidates for high-performance BES applications. Similarly, porous membranes and electrospun membranes, such as electrospun PVDF or GO-based membranes, offer lower resistance and higher ion permeability, allowing for improved BES efficiency.^6^ However, these membranes often face challenges related to fouling, structural degradation, and scalability.

The selection of an appropriate membrane plays a crucial role in optimizing BES and MFC efficiency. While commercially available membranes like Nafion remain a benchmark, researchers are actively developing cost-effective and performance-enhanced alternatives. Future advancements focus on reducing membrane costs, enhancing durability, and improving ion selectivity to develop scalable, long-lasting, and environmentally friendly BES technologies.^32,33^

### 2.1 Ion transport

Membranes are essential components in microbial fuel cells, ensuring the effective separation of the anode and cathode chambers while enabling controlled ion transport. This separation is critical for preventing direct mixing of the chambers, which could compromise the cell's performance by causing short circuits and reducing the overall efficiency of the system. The transport of ions through the membrane plays a key role in maintaining the necessary pH gradient for sustained electrochemical activity, as imbalances can lead to a loss of efficiency or even system failure.^12^

Selective ion transport is achieved using specialized membranes such as proton exchange membranes, cation exchange membranes, and anion exchange membranes. These membranes facilitate the migration of specific ions such as protons (H^+^), cations (*e.g.*, Na^+^, K^+^, Ca^2+^), and anions (*e.g.*, Cl^−^, SO_4_^2−^) between the chambers, enabling charge balance and sustaining redox reactions at the electrodes.^12^

Ion transport in membranes is governed by various mechanisms, including:

Diffusion: driven by concentration gradients, ions naturally move from a region of high concentration to low concentration.

Electrostatic migration: under an applied electric field, charged ions migrate toward the oppositely charged electrode.

Convection: the movement of solvent (*e.g.*, water) through the membrane can carry dissolved ions, contributing to overall ion transport.

In the bulk electrolyte, where concentration gradients are minimal, ion migration under an applied electric field predominantly drives current transport. The efficiency of this process depends on:

Membrane ionic conductivity: higher conductivity ensures minimal resistance to ion flow.

Transport number: this parameter quantifies the contribution of individual ions to the total current, directly impacting system efficiency.

Water uptake and swelling: proper hydration is necessary for maintaining membrane functionality, but excessive swelling can degrade mechanical stability.^34^

Furthermore, the performance of ion-transporting membranes can be affected by biofouling, where microbial growth leads to clogging and loss of function. Advanced membrane modifications, such as functionalized nanocomposites (*e.g.*, SPEEK/TiO_2_, GO-based membranes), ionic liquid-infused membranes, and hydrophilic polymer coatings, are being explored to improve ion selectivity, reduce crossover effects, and enhance membrane durability.

By enabling precise ion movement while maintaining chamber separation, membranes help preserve the electrochemical equilibrium, minimize internal resistance, and optimize electron transfer, making them indispensable to MFC technology. Future advancements in ionic conductivity, selectivity, and biofouling resistance will further enhance the role of membranes in improving MFC performance and scalability.^20,29^

### 2.2 Electron transport

Membranes are vital in MFCs for confirming operative electron transport by separating the anode and cathode chambers. This separation prevents electrons generated during microbial oxidation at the anode from directly migrating to the cathode through the electrolyte, which would lead to a short circuit. Instead, the electrons are channeled through an external circuit, enabling the controlled generation of an electric current.^25^ The cathode chamber typically supports oxygen reduction reactions, where oxygen acts as the primary electron acceptor. In many cases, abiotic cathodes utilize inorganic catalysts such as platinum; however, these materials exhibit reduced efficiency at the near-neutral pH conditions typical of MFCs. To address this limitation, research has focused on finding alternative oxygen-reduction catalysts, including activated carbon and advanced materials like graphene. Additionally, microbial and enzymatic cathodes have emerged as promising solutions for improving MFC performance. The oxygen reduction mechanism in MFCs is highly pH-dependent. Under neutral pH conditions, the reaction often generates hydroxide ions (OH^−^) rather than relying on protons, which are present in insufficient concentrations. This localized production of OH^−^ near the cathode surface can result in a pH increase, leading to the precipitation of cations such as calcium (Ca^2+^) and magnesium (Mg^2+^) as hydroxides. Such precipitation contributes to fouling, reducing the cathode's efficiency, particularly in saline or marine environments. By ensuring external electron flow and maintaining chamber separation, membranes optimize energy conversion and prevent undesirable electron leakage, thereby enhancing the overall functionality and efficiency of MFCs.^22^

### 2.3 Prevention of cross-contamination

In MFCs, membranes are essential components that block cross-contamination between the anode and cathode chambers, maintaining system stability and operational efficiency. Unwanted mixing, such as oxygen permeating into the anode or organic substrates migrating into the cathode, can severely impact the conditions needed for effective microbial and electrochemical processes.^35^ For example, oxygen infiltration disrupts the anaerobic environment crucial for microbial oxidation in the anode, while the movement of fuel to the cathode reduces the cell's energy output by bypassing the intended reactions. These membranes function as selective filters, permitting the transfer of specific ions necessary for balancing charge while restricting the movement of undesirable molecules. This selectivity ensures the preservation of distinct chemical conditions within each chamber, which is vital for maintaining the gradients required for consistent power production. Preventing cross-contamination is also critical to sustaining an anaerobic environment in the anode and avoiding fouling of the cathode by microbial growth or chemical deposits caused by migrating impurities. Innovative membrane technologies, such as ion-exchange membranes, are designed to further enhance the control of ion movement while minimizing the risk of contamination. Additionally, advanced configurations like separator-electrode assemblies integrate membranes directly between electrodes, reducing the distance for ion transfer and enhancing performance. By effectively managing cross-contamination, membranes ensure the reliable operation of MFCs, optimize energy generation, and support applications such as wastewater treatment, ion recovery, and water desalination, where chamber integrity is paramount.^36^

### 2.4 Enhancement of reactor performance

Membranes are integral components of MFCs, serving to separate the anode and cathode compartments while preserving their unique chemical environments. An effective membrane must ensure selective ion transport, block oxygen diffusion to the anode, and prevent the transfer of fuel and organic substances to the cathode. This separation is critical for sustaining electrochemical gradients, which are vital for maximizing power generation and optimizing reactor efficiency. Ion-exchange membranes, including cation-exchange membranes and anion-exchange membranes, are widely employed in MFCs due to their ability to regulate ion movement based on charge. AEMs are generally preferred when enhancing power output is a priority, while CEMs are more suitable for applications requiring higher coulombic efficiency (CE). The choice of membrane significantly impacts power density and the overall performance of the MFC. In addition to facilitating ion transport, membranes can boost MFC efficiency by concentrating substrates or ions near the electrode surfaces, thereby accelerating reaction kinetics. This localized enhancement improves the electrochemical reaction rates, contributing to increased power generation.

Membranes also play a pivotal role in mitigating biofouling on the cathode and enabling additional functionalities in MFCs, such as water desalination, ion removal, and wastewater treatment. Innovations like membrane-electrode assemblies (MEAs), where the membrane is directly integrated between the electrodes, have emerged as a means to reduce internal resistance and minimize energy losses by shortening the distance between electrodes. Despite these advancements, challenges persist, including the need to lower ion transport resistance and address pH imbalances caused by localized acidification or alkalinization. Enhancing membrane attributes including selectivity, permeability, and resistance to fouling are critical for overcoming these obstacles. While separator-less designs are being explored for their simplicity and applicability in waste treatment, membranes remain a cornerstone for achieving superior power output and advancing the multifunctionality of MFCs.^37^

## 3. Requirements for MFC membranes

The efficiency of MFCs is largely determined by the performance of their membranes, which are essential for effective energy production. Crucial membrane attributes, including selectivity, conductivity, and durability, significantly influence their functionality. Selectivity ensures the controlled movement of ions while preventing the passage of unwanted substances, conductivity enhances ion transport and minimizes resistance, and durability provides stability under challenging microbial and chemical environments. These characteristics collectively shape the membrane's role in maximizing MFC performance.^38,39^

### 3.1 Selectivity

The membrane's selectivity is a critical factor in the efficiency and durability of MFCs. The membrane must facilitate the targeted transport of ions, such as protons or hydroxide ions, while restricting the movement of unwanted substances and gases between the anode and cathode compartments. This controlled ion transfer is critical for maintaining the electrochemical gradient, which drives the electron flow necessary for generating electricity. To ensure optimal performance, the membrane must block the crossover of organic fuel molecules from the anode to the cathode, preserving fuel and efficiency. Additionally, it should prevent oxygen and other electron acceptors at the cathode from diffusing into the anode, maintaining the anaerobic conditions required for electroactive microbes that catalyze oxidation reactions. The membrane also plays a vital role in minimizing the transfer of harmful ions or compounds, such as sulphates, ammonia, and heavy metals, which could disturb the anodic microbial ecosystem. Effective selectivity reduces internal resistance and prevents issues like substrate crossover, pH fluctuations, and biofouling, all of which impact MFC performance. For ion-exchange membranes (IEMs), partially hydrophilic properties enhance ion transport while ensuring selective permeability. In contrast, porous membranes benefit from hydrophobic features to limit unwanted crossover. Chemical and microbial resistance further support the membrane's selectivity, ensuring stable and efficient operation of MFC systems.^36,37^

### 3.2 Conductivity

The ionic conductivity of a membrane is a pivotal factor influencing the performance and power output of MFCs. Efficient ion transport, such as protons or anions, between the anode and cathode compartments is crucial for maintaining electrochemical equilibrium, sustaining current flow, and minimizing internal resistance. Ion-exchange membranes (IEMs) benefit from partially hydrophilic characteristics, which create pathways that facilitate the movement of charged particles. On the other hand, porous membranes can function effectively with hydrophobic properties, utilizing their structural features to regulate ion transport. By ensuring effective ionic conduction, the membrane minimizes overpotentials related to ion transfer, enhancing the electron transfer rate and optimizing energy efficiency. Conductivity also plays an essential role in preserving a stable pH difference between the anode and cathode chambers. This stability is critical for the activity and survival of electroactive microbes, which are central to oxidation reactions at the anode. Insufficient conductivity may lead to pH imbalances, disrupting microbial function and negatively impacting system performance. Beyond ion transport, a membrane with high conductivity must also limit the diffusion of organic fuels, oxygen, or other electron acceptors that could disturb the anode environment. Such events can reduce energy recovery and decrease efficiency. Therefore, balancing excellent ionic conductivity with effective selectivity is essential for ensuring the optimal functionality of MFC membranes.^36,40,41^

### 3.3 Durability

MFC membranes need to withstand the severe conditions observed in microbial environments, such as exposure to alkaline or acidic solutions, mechanical stress, and biofouling. Durability ensures the MFC system's long-term stability and performance.^3,4^ Membranes play a vital role in determining the efficiency and durability of MFCs. Their ability to facilitate accurate ion transport, minimize internal resistance, and endure harsh operational conditions is critical for maintaining consistent performance and energy output. Advancing knowledge and improving these membrane properties are key to refining MFC systems, enabling them to serve as dependable and eco-friendly energy technologies that contribute to addressing current energy and environmental issues.

## 4. Fabrication and enhancement techniques for tailored polymeric membranes

Polymeric membranes with tailored properties play a vital role in advanced technologies such as MFCs, where their performance directly influences the efficiency and functionality of the system. A variety of fabrication techniques, including solution casting, electrospinning, and layer-by-layer assembly, offer distinct benefits in designing membranes with specific structural and functional attributes. Additionally, advanced methods such as phase inversion, template synthesis, and self-assembly provide enhanced control over membrane morphology and characteristics, enabling superior performance in specialized applications. To further enhance the capabilities of these membranes, techniques like surface modification, composite development, and nanostructuring are employed. These approaches improve key properties such as ion conductivity, mechanical strength, and selectivity, making the membranes highly adaptable to the demands of cutting-edge applications. By leveraging innovative fabrication and enhancement strategies, tailored polymeric membranes continue to evolve, addressing complex challenges in areas like energy production, environmental remediation, and separation technologies.^42–44^

### 4.1 Fabrication of tailored polymeric membranes

**Fig. 4:**
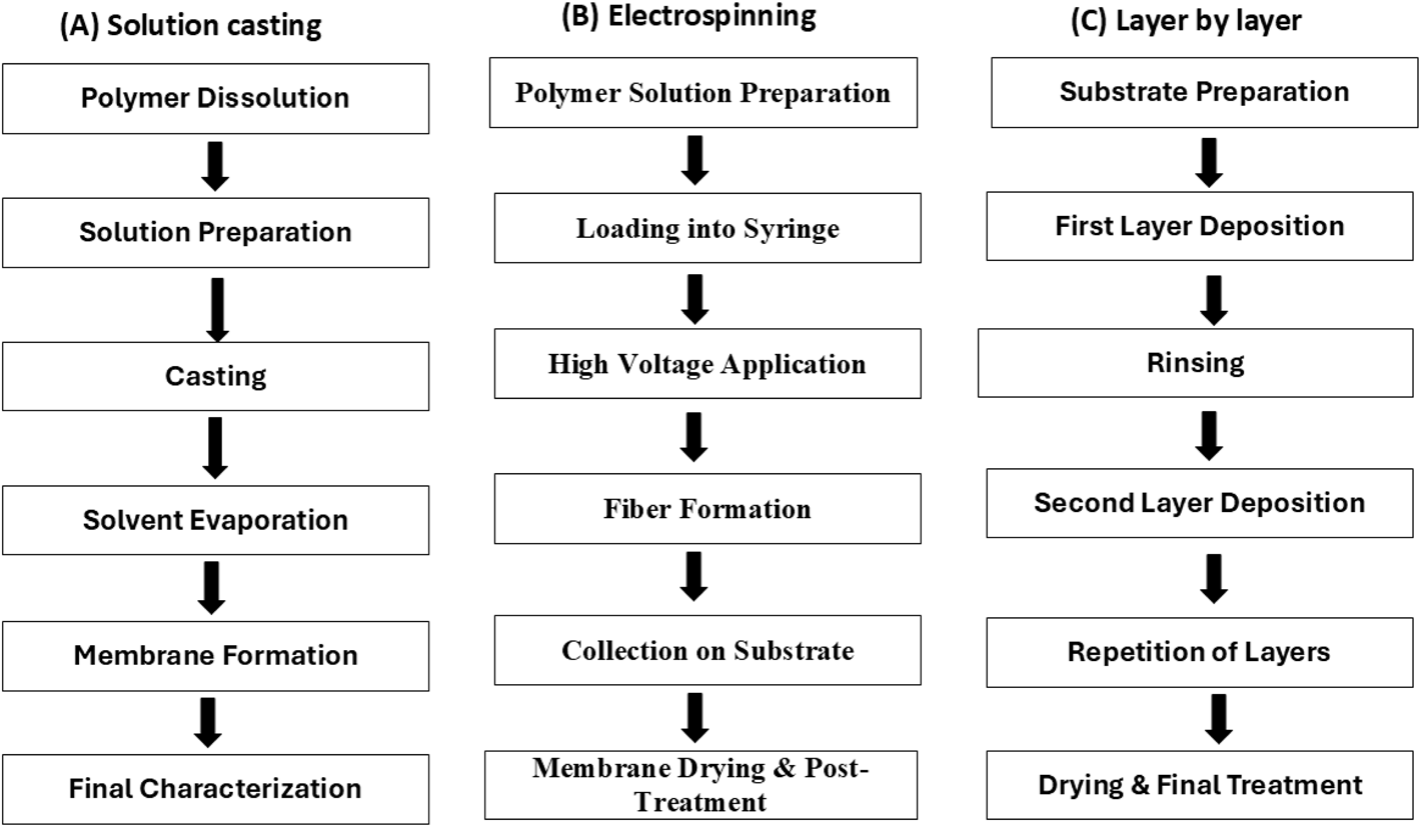
Schematic diagram of (A) solution casting, (b) electrospinning, (c) layer by layer.

The development of tailored polymeric membranes is essential for advancing cutting-edge technologies such as MFCs, where membrane performance is crucial for system optimization. Various fabrication methods, including solution casting, electrospinning, and layer-by-layer assembly, enable the creation of membranes with unique properties suited to specific applications. Solution casting is a straightforward and economical method for producing flat membranes, while electrospinning generates nanofibrous structures with enhanced surface area and ion conductivity. Layer-by-layer assembly offers meticulous control over membrane thickness and composition, allowing for highly specialized designs and the detailed schematic diagram are shown in Fig. 4. Moreover, sophisticated techniques like phase inversion, template synthesis, and self-assembly facilitate the production of membranes with intricate nanostructures and advanced functionalities. Together, these approaches empower the design of membranes tailored to meet the demands of modern applications, driving innovation in energy systems, environmental solutions, and separation processes.^43,44^

#### 4.1.1 Solution casting

In MFCs, solution casting is a widely used fabrication technique for tailored polymeric membranes. It provides a rather easy and economical way to create membranes with the required characteristics. Polymers such as polyvinylidene fluoride (PVDF), sulfonated poly(ether sulfone) (SPES), and sulfonated poly(ether ether ketone) (SPEEK) are dissolved in solvents such as dimethylformamide (DMF), dimethylacetamide (DMAc), or *N*-methyl-2-pyrrolidone (NMP). After the homogenous solution is produced, it is poured onto a mold or flat surface, and the solvent is allowed to evaporate, forming the membrane. Additives of any kind can be used during solution casting to improve the selectivity, conductivity, and durability of the membrane. It is restricted to basic or flat geometries, though, and casting and evaporation conditions must be carefully controlled. Although solution casting has its limits, it is nevertheless a useful method for creating polymeric membranes customized for certain MFC applications. Research on the development and operation of solution-cast membranes in MFCs is provided by Ayyaru and Dharmalingam (2011)^20^ and Mahendiravarman and Sangeetha (2013).^45^ These studies emphasize the significance of material selection and processing factors in obtaining ideal membrane performance.

#### 4.1.2 Electrospinning

A versatile and efficient method for creating tailored polymeric membranes for a range of uses is electrospinning. A high voltage is supplied to a polymer melt or solution during electrospinning, causing a charged jet of polymer to be pulled in the direction of a grounded collector. The solvent evaporates as the jet moves, depositing a nanofibrous membrane on the collector. High surface area-to-volume ratios, adjustable pore diameters, and regulated fiber orientations may all be achieved with this technique, which improves mass transfer and ion conductivity in MFCs. Poly(vinyl alcohol) (PVA), poly(ethylene oxide) (PEO), and polyacrylonitrile (PAN) are typical polymers utilized in electrospinning for MFC applications. The efficacy of electrospun membranes in enhancing the power output and performance stability of MFCs has been proven in study conducted by Enamala *et al.*, 2020.^6^ These findings emphasize the promise of this fabrication process for the development of advanced membrane designs.

#### 4.1.3 Layer-by-layer assembly

Layer-by-layer (LbL) assembly is a new technology for manufacturing specialized polymeric membranes with precise control over thickness, composition, and functionality, making it appropriate for MFC applications. In LbL assembly, oppositely charged polyelectrolytes or functional nanoparticles are successively deposited onto a substrate, generating a multilayered membrane. This technique enables the improvement of membrane characteristics including ion conductivity, selectivity, and mechanical strength by incorporating different functional elements, such as conductive polymers or metal nanoparticles. Commonly utilized polymers in LbL assembly for MFC membranes are poly(diallyldimethylammonium chloride) (PDDA), poly(ethyleneimine) (PEI), and poly(sodium 4-styrenesulfonate) (PSS). Study conducted by Vajihe *et al.*, (2018)^9^ has shown how LbL-assembled membranes can improve the stability and power output of MFCs, indicating that this fabrication approach has the potential to be used in MFCs with advanced membrane design.

#### 4.1.4 Other advanced techniques

Several different cutting-edge methods are being investigated in addition to solution casting and electrospinning for the creation of customized polymeric membranes for MFCs. Phase inversion, template synthesis, and self-assembly are some of these methods. By altering the solvent/non-solvent ratio, phase inversion causes a polymer to precipitate from a homogenous solution and creates a porous membrane structure. Through template synthesis, distinct nanoporous structures in the membrane are produced using sacrificial templates. A controlled nanostructure can be formed in membranes by the process of self-assembly, which is based on molecules spontaneously organizing into organized structures like vesicles or micelles. These cutting-edge methods provide exact control over the composition and characteristics of the membrane, which may improve MFC performance.^40^

The fabrication of tailored polymeric membranes involves diverse techniques that provide a wide range of design possibilities to meet specific performance requirements in applications like microbial fuel cells. Solution casting, while simple and cost-effective, is limited to basic membrane geometries and may not offer the fine control required for high-performance applications. On the other hand, electrospinning produces nanofibrous membranes with significantly higher surface area and enhanced conductivity, making them more suitable for applications where mass transfer and ion conductivity are crucial. Layer-by-layer assembly provides a finer level of control over membrane composition and thickness, enabling the creation of membranes with multiple functional layers tailored for advanced uses. In comparison, advanced techniques like phase inversion and self-assembly allow for the development of membranes with complex nanostructures and properties that are challenging to achieve with simpler methods. Overall, each fabrication method offers unique benefits, with electrospinning and layer-by-layer assembly emerging as the most versatile for high-performance membrane applications.

### 4.2 Enhancement techniques of tailored polymeric membranes

The enhancement of tailored polymeric membranes is of paramount importance for advancing their functional performance in microbial fuel cells (MFCs) and broader bioelectrochemical systems (BES). Membranes used in these systems must exhibit a combination of high ionic conductivity, mechanical robustness, chemical stability, selective ion transport, and resistance to biofouling. To meet these multifaceted requirements, several advanced techniques have been developed, including surface modification, composite membrane fabrication, and nanostructuring approaches.

Surface modification techniques—such as plasma treatment, chemical grafting, UV irradiation, and the application of functional coatings—are widely employed to tailor membrane surface characteristics without altering bulk properties. These modifications introduce functional groups (*e.g.*, –SO_3_H, –NH_2_, –NR_4_^+^), which enhance hydrophilicity, ion exchange capacity, and antifouling properties. Plasma treatment, for instance, improves surface energy and functional group density, thus facilitating better ion mobility and microbial compatibility. Chemical grafting allows selective incorporation of ionic moieties to enhance conductivity and stability under operational conditions.

Composite membrane fabrication involves blending polymer matrices with functional fillers such as metal oxides (*e.g.*, TiO_2_, SiO_2_, Fe_3_O_4_), layered silicates (*e.g.*, montmorillonite), and carbon-based nanomaterials (*e.g.*, graphene oxide, multi-walled carbon nanotubes). These fillers not only improve the mechanical strength and thermal stability of the membranes but also significantly enhance ionic conductivity and chemical resistance. For example, SPEEK/TiO_2_ composites offer improved oxidative stability and conductivity, while chitosan/MWCNT composites provide a synergistic improvement in bio-compatibility and electron transfer. The incorporation of fillers can also mitigate swelling behavior and minimize oxygen crossover, thereby extending membrane lifespan in MFC environments.

Nanostructured membranes, fabricated using techniques such as electrospinning, layer-by-layer (LbL) assembly, and template-assisted synthesis, provide high surface-area-to-volume ratios, tunable porosity, and improved interfacial contact with microbial communities. Electrospun membranes, characterized by interconnected nanofibrous networks, facilitate enhanced ion transport and water retention. The LbL technique enables precise control over membrane thickness and composition through the alternate deposition of oppositely charged species, resulting in membranes with high selectivity and excellent antifouling properties. Template-assisted methods can be employed to engineer well-ordered pore architectures, improving mass transfer and reducing internal resistance.

Each enhancement strategy contributes uniquely to improving membrane performance in MFCs. Surface modifications offer a simple and scalable means of enhancing membrane functionality, yet their effects may be confined to the surface and degrade with time. Composite membranes strike a balance between performance and durability but pose challenges in achieving homogeneous dispersion and long-term filler stability. Nanostructured membranes provide exceptional control over ion transport and interface behaviour, although their fabrication is often complex and less amenable to large-scale production.

Ultimately, the selection of an appropriate enhancement technique depends on the specific operational requirements of the MFC system. For instance, high-performance lab-scale MFCs may benefit from multifunctional nanocomposite or nanostructured membranes, whereas cost-sensitive, large-scale systems may prioritize surface-modified or polymer-blend membranes for economic viability. Future directions point toward the integration of these strategies, aiming to develop hybrid membranes that synergistically combine the benefits of surface functionalization, structural reinforcement, and nanoscale architecture.

These enhancement techniques collectively open new avenues for tailoring membranes that meet the evolving demands of sustainable energy generation, environmental remediation, and advanced separation technologies.^24–26^

#### 4.2.1 Surface modification techniques

Surface modification techniques are widely adopted to improve the physicochemical properties of polymeric membranes for microbial fuel cells (MFCs) and other bioelectrochemical applications. These techniques aim to enhance critical membrane parameters such as selectivity, ionic conductivity, antifouling behaviour, and long-term stability by modifying only the surface layer without compromising the bulk mechanical integrity of the membrane.

Among the most commonly employed techniques are plasma treatment, chemical grafting, and functional coating deposition.^43,44^ Plasma treatment involves exposing the membrane surface to a low-temperature plasma, typically generated using gases such as oxygen, nitrogen, or argon. This process introduces reactive species (*e.g.*, ions, radicals, and UV photons) that break chemical bonds on the polymer surface, creating active sites and incorporating new functional groups such as –OH, –COOH, and –NH_2_. These modifications significantly increase surface energy, hydrophilicity, and interfacial adhesion, thereby improving ion transport and biofilm compatibility. Plasma treatment is considered a clean and rapid technique, and its intensity and exposure time can be precisely controlled. However, its effects are often limited to the uppermost nanolayers of the surface and may diminish over time due to surface relaxation or contamination.

Chemical grafting introduces desired functional groups onto the membrane surface by covalently bonding monomers or polymers through free-radical or ionic mechanisms. Commonly grafted functionalities include sulfonic acid (–SO_3_H) groups for proton conduction or quaternary ammonium groups (–NR_4_^+^) for anion exchange capability. Grafting can be initiated thermally, photochemically, or by using plasma-activated surfaces, and provides greater chemical stability and permanence of the modification compared to plasma treatment alone. While grafting offers good control over surface chemistry, it often requires the use of solvents and initiators, making it a more complex and less environmentally friendly option.

Functional coating deposition is another versatile method, involving the application of ultrathin layers of materials with specific functionalities.^46,47^ For example, coating membranes with conducting polymers such as polyaniline (PANI) or polypyrrole (PPy) improves surface conductivity and facilitates electron transfer. Similarly, the deposition of inorganic nanoparticles such as TiO_2_, SiO_2_, or GO (graphene oxide) can enhance surface hydrophilicity, ion selectivity, and antifouling behaviour. These coatings can be applied through dip-coating, spin-coating, spray-coating, or layer-by-layer (LbL) assembly methods. While coatings offer the flexibility of introducing multifunctional properties, challenges such as coating adhesion, mechanical delamination, and long-term stability under operational conditions must be addressed.

Each surface modification technique offers distinct advantages and limitations. Plasma treatment is rapid and solvent-free but typically results in shallow modifications that may fade over time. Chemical grafting, although more complex, ensures durable chemical changes and can be tailored to introduce highly specific functionalities. Functional coatings provide the opportunity to integrate novel materials with diverse functionalities, but they require careful optimization to avoid compromising membrane permeability or structural stability.

The selection of the appropriate modification method depends on the target membrane property and operational environment. For instance, plasma treatment may be suitable for enhancing surface energy and biofilm adhesion in short-term or disposable MFC devices, whereas grafting is better suited for membranes requiring long-term chemical stability. Functional coatings, especially those incorporating nanomaterials or redox-active species, are ideal for applications that demand multifunctional performance, such as simultaneous proton conduction and antifouling activity. In practice, combining these techniques (*e.g.*, plasma-assisted grafting or coating over grafted surfaces) has shown synergistic effects and holds promise for developing next-generation, high-performance membranes for sustainable energy and environmental applications.

#### 4.2.2 Composite membranes

Composite membranes have gained significant attention in recent years as a powerful approach to enhance the performance of polymeric membranes, particularly in microbial fuel cell (MFC) applications. These membranes are engineered by integrating two or more materials—typically a polymer matrix and functional fillers—to leverage the combined advantages of each component while addressing limitations associated with conventional single-component membranes.^48^ The primary objectives of composite membrane design in MFCs include enhancing proton conductivity, mechanical strength, chemical and thermal stability, and resistance to fouling.^20,49–51^ Common fabrication strategies include polymer–polymer blending, incorporation of inorganic or carbon-based nanoparticles, and functionalization with ionic moieties. For instance, blends such as SPEEK/PES or PVA/PAA are used to balance hydrophilicity and mechanical durability. Incorporation of fillers such as TiO_2_, SiO_2_, Fe_3_O_4_, MMT, and Zr-based MOFs enhances structural integrity and ion transport. Additionally, carbon nanomaterials like graphene oxide (GO) and multi-walled carbon nanotubes (MWCNTs) improve the electrical conductivity and biofilm compatibility of membranes. Functionalization with sulfonic acid (–SO_3_H) or quaternary ammonium (–NR_4_^+^) groups can significantly improve ion exchange capacity and selectivity.

The use of composite membranes has proven particularly effective in reducing biofouling and improving long-term operational stability in MFCs. For example, the integration of antimicrobial nanoparticles such as silver (Ag), zinc oxide (ZnO), or TiO_2_ provides inherent resistance against microbial colonization, thereby prolonging membrane life and maintaining electrochemical performance. Moreover, fillers like montmorillonite (MMT) and metal–organic frameworks (MOFs) offer additional water retention and ion conduction pathways, reducing internal resistance and enhancing proton transport efficiency. From a mechanical standpoint, reinforcing polymer matrices with nanoparticles significantly enhances tensile strength, dimensional stability, and swelling resistance, which are critical under fluctuating hydration and pH conditions typically encountered in MFC operations. The composite structure also enables membranes to better withstand oxidative degradation, a common issue in BES.

Despite these advantages, several challenges persist. Achieving homogeneous dispersion of nanoparticles within the polymer matrix remains critical, as agglomeration can lead to performance inconsistencies and structural defects. Moreover, the compatibility between the filler and the polymer matrix must be optimized to ensure efficient ion transport and structural cohesion. Comparatively, polymer–polymer blends offer ease of processing and cost-effectiveness but may face limitations in ionic conductivity. Nanoparticle-filled composites provide superior performance characteristics but require careful optimization of filler content and dispersion methods. Functionalized membranes, while offering targeted enhancements, may involve more complex synthesis procedures and higher production costs. Overall, composite membranes represent a highly tuneable and promising class of materials for MFC applications. By strategically combining material functionalities, these membranes can meet the stringent requirements of ion selectivity, conductivity, stability, and fouling resistance. Future research is expected to focus on the development of multifunctional composite systems, bio-based fillers, and scalable fabrication techniques, aiming to bridge the gap between laboratory performance and real-world applicability in sustainable energy technologies.

#### 4.2.3 Nanostructured membranes

Nanostructured membranes represent a highly promising advancement in polymer membrane technology, particularly within the context of MFCs and other BES. Their unique nanoscale architecture imparts several advantageous characteristics, including enhanced surface area, tuneable porosity, improved ionic conductivity, and superior mechanical integrity.^2,46,47^ These features collectively contribute to improved ion transport, reduced internal resistance, and increased resistance to biofouling key factors that dictate membrane performance in electrochemical energy conversion systems. A variety of fabrication strategies have been employed to develop nanostructured membranes. Among these, electrospinning has been widely utilized to generate nanofibrous membranes characterized by interconnected porous networks, high surface area-to-volume ratios, and excellent water retention capabilities. Such properties facilitate efficient ion migration and contribute to sustained electrochemical activity. Additionally, the nanofibrous structures produced *via* electrospinning have demonstrated favorable microbial adhesion and biofilm development, which are critical for improving extracellular electron transfer in MFCs.

Another widely adopted technique is layer-by-layer (LbL) assembly, which allows for precise and sequential deposition of oppositely charged polyelectrolytes or functional nanoparticles onto a substrate. This method enables atomic-level control over membrane thickness, composition, and surface chemistry, thereby allowing the fabrication of highly selective, chemically resistant, and antifouling membrane surfaces. LbL-assembled membranes have shown exceptional promise in BES due to their stability and tunability under diverse operating conditions. Template-assisted synthesis is also employed to fabricate membranes with ordered and uniform nanoporous structures. By using soft or hard templates during membrane formation, it is possible to produce membranes with well-defined pore sizes and geometries that promote uniform mass transport, minimize diffusion resistance, and ensure selective ion passage. These features are highly advantageous for maintaining consistent electrochemical performance and prolonging membrane life in MFC applications.

In addition to nanostructuring, two other major membrane enhancement approaches—surface modification and composite membrane fabrication—are commonly used to improve membrane function. A comparative evaluation of these methods reveals their complementary nature and distinct performance impacts. Surface modification techniques, including plasma treatment, chemical grafting, and functional coatings, are primarily focused on modifying the membrane interface to improve hydrophilicity, ion exchange capacity, and fouling resistance. These modifications are generally confined to the surface and may degrade over time due to operational stresses, offering only limited influence on bulk properties such as mechanical strength and internal conductivity.

In contrast, composite membranes, developed by embedding inorganic or carbon-based nanofillers (*e.g.*, TiO_2_, SiO_2_, GO, MWCNTs, MOFs) into polymer matrices, provide comprehensive performance enhancements. These include increased mechanical stability, improved thermal and chemical resistance, and elevated ionic conductivity. However, challenges such as filler agglomeration, phase separation, and fabrication complexity must be addressed to ensure uniform performance and scalability. Nanostructured membranes, owing to their high surface area and tuneable pore architecture, offer superior ion transport properties and reduced mass transfer limitations. Their structural versatility allows for integration with other enhancement techniques, including surface functionalization and composite incorporation, to create hybrid membranes with multifunctional properties tailored to specific electrochemical environments.

Nanostructured membranes, through techniques such as electrospinning, LbL assembly, and template synthesis, present transformative opportunities in the design of high-performance membranes for MFCs and related applications. When compared to surface-modified and composite membranes, nanostructured membranes offer distinct advantages in terms of ion conductivity, mass transport, and fouling resistance. Future efforts should focus on the development of scalable, cost-effective fabrication methods and the integration of multifunctional materials to create next-generation hybrid membranes capable of meeting the rigorous demands of sustainable energy and environmental systems.

### 4.3 Role of conducting polymers in microbial fuel cells (MFCs)

Conducting polymers such as polyaniline (PANI), polypyrrole (PPy), and poly(3,4-ethylenedioxythiophene) (PEDOT) play a vital role in enhancing MFC performance by improving electron transfer, ion conductivity, and mechanical stability. These polymers act as electrode coatings that facilitate microbial adhesion and promote direct electron transfer between bacteria and the electrode surface, thereby improving electrochemical activity and power generation. Their inherent high electrical conductivity makes them excellent materials for anode and cathode modifications, reducing internal resistance and enhancing overall MFC efficiency.

Additionally, conducting polymer composites incorporating carbon nanomaterials (*e.g.*, graphene oxide (GO), carbon nanotubes (CNTs)), metal oxides, and biopolymers significantly improve catalytic activity, durability, and mechanical strength. Such composites not only facilitate faster charge transfer but also enhance proton and ion exchange, improving the ionic conductivity of membranes used in MFCs. Conducting polymer-based membranes, such as PANI-modified proton exchange membranes, have demonstrated superior ionic selectivity, reduced oxygen crossover, and enhanced antifouling properties, which contribute to the long-term stability of MFC operations.

Moreover, the use of conducting polymers in cathode materials has been shown to enhance oxygen reduction reactions (ORR), replacing expensive metal catalysts such as platinum. The synergistic effect of conducting polymers with metal-free catalysts or biocatalysts offers a sustainable and cost-effective solution for MFCs. These advancements have led to significant improvements in power output, coulombic efficiency, and operational stability, making conducting polymers a promising material class for next-generation bioelectrochemical energy systems.^52^

## 5. Characterization methods for polymeric membranes

Recent progress in the characterization of polymeric membranes has greatly improved their suitability for high-performance applications, including MFCs. Advanced analytical methods now facilitate the accurate assessment of essential membrane attributes such as ion exchange capacity (IEC), water absorption, dimensional swelling, and electrochemical properties. Techniques like Fourier-transform infrared (FTIR) spectroscopy, electrochemical impedance spectroscopy (EIS), and dynamic mechanical analysis (DMA) offer valuable insights into ion transport mechanisms, hydration dynamics, and structural resilience under challenging conditions. These state-of-the-art methods have deepened our understanding of membrane functionality, enabling the development of membranes with enhanced efficiency and durability. By capitalizing on these advancements, scientists can fine-tune membrane properties to achieve exceptional ion selectivity, conductivity, and mechanical strength, ensuring dependable performance in MFCs.^53,54^ Ultimately, these characterization outcomes support the creation of membranes that align with the rigorous demands of contemporary energy and environmental applications.

### 5.1 Ion exchange capacity (IEC)

The characterization of polymeric membranes used in MFCs and other applications requires the measurement of ion exchange capacity (IEC). One unit of weight or volume of the membrane equals one thousand exchangeable ions, or IEC. It's calculated by taking a measurement of the amount of base or acid needed to balance the membrane's fixed ionic groups. For applications requiring ion selectivity and conductivity, the IEC value offers information on the membrane's ion transport and exchange properties.^36^ To determine the IEC, several techniques can be applied, including titration, conductometric titration, and Fourier-transform infrared (FTIR) spectroscopy.

### 5.2 Water uptake and swelling behaviour

Polymeric membranes need to be characterized by their water uptake and swelling behaviour, especially for applications like microbial fuel cells (MFCs) where performance can be greatly impacted by hydration levels. The ability of a membrane to absorb water affects its mechanical characteristics, ion conductivity, and fouling behaviour. This is known as water absorption. On the other hand, swelling behaviour defines how a membrane's dimensions change in response to water or other solvents, influencing the size of its pores and the structure of the membrane. A common method for determining these characteristics is to immerse the membrane in a solvent and track changes in weight or thickness over time.^36^

### 5.3 Chemical and mechanical stability

Polymeric membranes must have both chemical and mechanical stability, particularly in high-stakes applications such as MFCs. Chemical stability describes a membrane's capacity to withstand deterioration in extreme conditions, like the acidic or alkaline conditions found in MFCs. Conversely, mechanical stability refers to a membrane's capacity to hold together structurally in the face of variations in pressure or mechanical stress. These characteristics are frequently assessed using a variety of techniques, including as dynamic mechanical analysis (DMA), tensile strength testing, and immersion experiments in harsh solutions.^41^

### 5.4 Electrochemical impedance spectroscopy (EIS)

An effective method for assessing the electrical characteristics of polymeric membranes is electrochemical impedance spectroscopy (EIS), especially in the context of microbial fuel cell applications. By measuring a membrane's impedance at various frequencies, EIS can provide important details regarding its double-layer capacitance, charge transfer resistance, and ionic and electronic conductivity. This method consists of applying an AC signal with a modest amplitude on the membrane and examining the impedance spectra that results. Understanding ion transport pathways and identifying variables influencing MFC performance are two ways that EIS can aid in optimizing membrane design.^26^

The characterization of polymeric membranes has significantly advanced, offering critical insights into their properties and enabling their enhancement for applications such as MFCs. Different analytical techniques provide essential information about key performance parameters. Ion exchange capacity (IEC) is evaluated through methods like titration and FTIR spectroscopy, which determine the membrane efficiency in facilitating ion transport. Studies on water uptake and swelling behaviour, often conducted through immersion experiments, reveal how hydration impacts mechanical strength, ion conductivity, and membrane morphology. Chemical and mechanical stability is assessed using tools like dynamic mechanical analysis (DMA) and tensile strength tests, which highlight the membrane's resilience to chemical and physical stresses in demanding environments. Electrochemical impedance spectroscopy (EIS) further contributes by offering detailed insights into ionic conductivity, charge transfer resistance, and ion transport pathways. Together, these techniques provide a comprehensive understanding that drives the development of membranes with superior performance, durability, and efficiency for modern energy applications.

## 6. Separator components used in membrane exchange

The effectiveness of MFCs is heavily dependent on the membranes used to separate the anode and cathode chambers. These membranes facilitate ion movement, manage pH gradients, and prevent undesirable crossover, thus influencing overall performance. Various membrane types, such as cationic, anionic, bipolar, and porous membranes, each offer unique characteristics that impact efficiency. Furthermore, membraneless MFC configurations have gained attention due to their potential to reduce internal resistance and biofouling. The choice of membrane plays a crucial role in optimizing MFC functionality and addressing operational challenges.^55,56^

### 6.1 Cationic exchange membrane (CEMs)

The ideal separators for MFCs have traditionally been cation exchange membranes, such as Nafions, Hyflons, Zirfons, and CMI 7000. This is so that protons generated in the anode chamber can be easily conducted by CEMs into the cathode chamber. The Nafions, the most used CEM in MFCs, have fluoro carbon backbone with sulfonate group attachment, which improves proton transport through it.^50,51^ Higher maximum voltage (670 mV), current density of 150.6 mA m^−2^, and power density of 31.32 mW m^−2^ were achieved by MFCs employing thinner Nafion 112 membranes. This is because the CE of MFCs is greatly reduced by thinner membranes, which, however, promote substrate and oxygen permeability.^35,57^ Furthermore, Nafion 117 and Ultrexs CMI-7000 membrane performance is about similar. Thin Hyflons, a CEM containing perfluoro sulfonyl fluoride vinyl ether membranes, produce higher mean voltages, mean current densities, and mean power densities than thick Hyflons membranes in MFCs with oxygen diffusion cathodes,.^58^ This is because thin membranes have low membrane resistance, like Nafion.

Further research demonstrated that the performance of the MFC stack utilising Hyflons was significantly worse than that of the same one using regular PEM.^36^ This result is unreliable, however, because the low performance could also be the result of fuel deficiency, which causes a decrease in bacterial activity.^59^ Zirfons is an additional ultra-filtrated composite material that is made of an asymmetrical polysulfone membrane structure with ZrO_2_ filler particles.^20^ It displayed a higher oxygen mass transfer coefficient (1.9 × 10^3^ cm s^−1^) than Nafions 117 (2.8 × 10^4^ cm s^−1^), as well as a lower specific ionic resistance (2727 Ω cm) than Nafions 117 (17 000 Ω cm). Another CEM, Fumaseps, had ionic resistance comparable to Nafion 117 (16 000 Ω cm) due to its high mean pore size, which favored higher ion transfer and oxygen diffusion. When the membranes were filled with hydrogel, tubular MFCs with air cathodes and CMI-7000, a CEM in MEA, recorded a respectably high voltage and power density.^37^ Nafion and other CEMs employed as separators in MFCs found problems related to proton accumulation in the anode chamber,^17^ pH splitting between the anode and cathode chambers, oxygen transfer from the cathode to the anode chamber, substrate loss, and biofouling.^60^

### 6.2 Anionic exchange membrane (AEMs)

The necessity for a CEM replacement and challenges like ionic splitting resulted in the research on AEMs that could act as proton carriers and transport hydroxide anions to the anode from the cathode chamber.^40^ AEM uses a cutting-edge method called proton transfer mechanism that enhances MFC performance by preventing proton accumulation in the anode chamber. In fact, this material's pH splitting (pH = 0.27) is 7 times lower than Nafion 117's (pH = 1.8).^61^ In single-chamber MFCs, it is frequently noticed that AEMs and CEMs twist after a number of operating cycles. As a result of the greater voids in AEMs compared to CEMs, the excess pH solution near the voids decreased MFC performance.^62^ However, there was an improvement in AEM performance after suppressing the AEM-MEA, and that improvement was bigger than that of the similarly treated CEM.^62^

Commercially available membrane (AFN), a low resistance membrane with the maximum current density (0.38 mA cm^−2^), high oxygen mass transfer coefficient (1.26 × 10^−4^ cm s^−1^), and high oxygen flow (3.0 × 10^−8^ mmol s^−1^ cm^−2^) among the commercially available AEMs, is regarded as the best. For AM-1, the comparable numbers are 0.28 mA cm^−2^, 0.98 × 10^−4^ cm s^−1^, and 2.3 × 10^−8^ mmol s^−1^ cm^−2^. The corresponding values for ACS are 0.21 mA cm^−2^, 0.65 × 10^−4^ cm s^−1^, and 1.6 × 10^−8^ mmol s^−1^ cm^−2^.^63^

### 6.3 Bipolar membrane (BM)

The BPM is an assembly of a series connection of two selective layers, one each for cationic and anionic species. The transition region is the area that lies between the layers that exchange cations and anion.^64^ BPM allows for the instantaneous protons and hydroxide ions migration, which are created in the transition area when water molecules split. BPMs have been applied to MFC applications.^64–66^ The use of BPM was shown to maintain the lowest pH at the cathode in long-term operation, despite water splitting processes permitting a slow pH shift between 2 and 2.5. On the other hand, an MFC utilising Nafion 117 observed a rapid rise in catholyte in their study. Shabani *et al.*, 2009 (ref. 65) and Rahimnejad *et al.*, 2011 (ref. 66 and 67) also noted that the BPM had the lowest performance (*i.e.* cumulative bio hydrogen production) among the four different types of IEMs. However, Rahimnejad *et al.*, 2012 (ref. 56) and Ghangrekar 2007 (ref. 60) observed that electrolyte ion migration to the transition area on a large scale prevented BPM from controlling the pH gradient in MFCs. Therefore, it might not be viable to eliminate the pH gradient in BES using BPM.

### 6.4 Porous membranes

There is evidence that using affordable porous membranes, such glass wool, can lower the cost of electricity generation and wastewater treatment^45^ and micro filtrated membranes,^32^ are employed as separators in MFCs. The de-colorization of azo dyes is one prominent use of micro filtrated membranes as separators, in which the porous membrane structure allows diffusion of oxygen, degradation of the intermediates produced by azo bond breaking.^32^ Compared to membraneless MFCs, porous membranes fall short of completely stopping the crossover. The main benefit of a porous membrane is its initial low internal resistance, but this too rises over time owing to biofouling.^32^

### 6.5 Membraneless MFCs

The main downsides such as fouling due to biological sources, could be eliminated, resulting in membrane free MFCs, which are attributed with low internal resistance.^68^ The CE reduces to 20% greater than MFCs with membranes in membraneless MFCs due to a high proton transfer rate that causes a strong oxygen diffusion towards the anode.^41,57^ Additionally, in membraneless MFCs, the development of biofilms on the cathode prevents oxygen from diffusing to the cathode, which lowers MFC performance.

One membraneless MFC design forces electrolytes to continuously flow from the anode compartment to the cathode compartment, enhancing proton flow while limiting oxygen diffusion in the other direction. This significantly reduces oxygen diffusion from the cathode compartment to the anode compartment.^57^ Although the membraneless MFC's COD removal effectiveness could reach up to 90.5%, the majority of it happens in the cathode compartment due to aerobic bacteria.^69^ Membrane biofouling difficulties, a lack of membrane internal resistance, cheaper operating costs, and others are some of the undeniable benefits of membraneless technology.^17^ The selection of membrane material is a key factor in optimizing MFC performance. Each membrane type, from CEMs and AEMs to porous and membraneless designs, brings its own benefits and drawbacks, affecting ion transport, fouling resistance, and operational stability. By advancing membrane technology and refining design approaches, MFC systems can be improved to offer more sustainable and cost-effective energy solutions, enhancing their viability for environmental and energy applications.

## 7. Factors influencing separator efficiency

The efficiency of MFCs is heavily impacted by factors such as membrane resistance, oxygen leakage, substrate migration, and pH imbalances. These issues disrupt critical processes, including ion transfer, microbial activity, and redox reactions, leading to reduced energy output. Membrane resistance limits ion flow, while oxygen and substrate crossover cause energy losses and biofilm formation. Additionally, pH variations hinder microbial performance. Advancing membrane design and refining operational strategies are crucial steps toward addressing these challenges and maximizing MFC performance.^70,71^

### 7.1 Resistance across the membrane

An MFC's internal resistance, which is made up of the resistances of its anode, cathode, electrolyte, and membrane, makes up a significant portion of its overall resistance.^29,72^ Due to the limitation of the proton diffusion from the anode to the cathode, MFCs with high internal resistance perform poorly.^73^ MFCs using porous low resistance membranes, such as micro filtrated membranes, perform poorly because of the extensive oxygen and substrate crossover *via* the membrane pores, which reduces the CE and power density.^74^ Despite having lower internal resistance than non-porous membranes, the use of porous membranes as separators in MFC is generally discouraged due to their high rates of oxygen and substrate crossover.^75^ The electrolyte concentration and composition are proven to have an impact on membrane resistance. Ohmic resistance, which makes up the majority of the membrane resistance in MFCs, can be utilized to calculate the total membrane resistance.^12,32^ It has been revealed that using a ion conductive membrane that facilitates ion transit results in higher current and power densities.

### 7.2 Oxygen diffusion

Another significant problem with MFCs is diffusion of oxygen between the electrode compartment because the overall performance is hampered by voltage loss brought on by an increase in redox potential as a result of aerobic bacteria consuming substrate instead of anaerobic bacteria.^76^ Considering that oxygen is the most fervent electron acceptor, it almost completely depletes the anode, lowering the MFC's coulombic efficiency (CE). However, the decrease in MFC performance caused by oxygen diffusion is just temporary and will quickly be reversed by anaerobic bacterial activity.^77^

Due to the nature of porous membranes, oxygen diffuses through the pores more quickly than in the case of non-porous membranes. With an oxygen mass transfer coefficient of *K*_o_ = 1.3 × 10^4^ cm s^−1^, the Nafion membrane, the most popular non-porous membrane in MFC, is shown to be just little permeable to oxygen.^3,78^ Considering that there are basically no physical obstacles to stop oxygen penetration into the anode compartment, membraneless MFCs have the maximum oxygen diffusion rate. As a result, its CE was found to be over 20% lower than that of MFCs with membranes.^59^

According to Kim *et al.*, 2012 (ref. 41) the Ultrex CMI-7000 membrane performed on par with Nafion. The power density and CE of an MFC employing Nafion were 514 mW m^−2^ and 41–46%, whereas they were 480 mW m^−2^ and 41–54% for an MFC utilising CMI-7000. It has been determined that Selemion, a hydrocarbon type PEM created by the Japanese company Asahi Glass Co., is a good substitute for Nafion 117. It has lower internal resistance, reduced oxygen permeability, and is less expensive. Lefebvre *et al.* found that an MFC running on Selemion had a 25% higher power density than one running on Nafion.

### 7.3 Substrate crossover

Membranes can also allow for the diffusion of substrates in wastewater in the same way that oxygen does, moving from the anaerobic anode compartment to the aerobic cathode compartment, or in the exact opposite direction of how oxygen diffuses. Large pore size porous membranes favour substrate crossover more frequently than AEM.^79^ Acetates, butyrates, and propionates, which are negatively charged substrates,^80^ diffuse through solid membranes more slowly in non-porous AEMs. Aerobic bacteria oxidise the substrates as they reach the cathode chamber, producing additional electrons for the ORR at the cathode and resulting in a short circuit within the compartment lowering CE.^17,33,81^ Biofouling is the term used to describe the process when aerobic bacteria produce a biofilm on the cathode surface as a result of substrate crossing.^59,82^ In fact, bio-fouling temporarily increases the power density of MFCs at startup. However, since progressively growing biofilm restricts oxygen transport to the cathode and the active surface area of the cathode accessible for ORR, the power density decreases noticeably over time.^32^ In order to generate power continuously, substrate crossover rate must be low.

### 7.4 pH splitting

MFCs performance is affected by the phenomena wherein membrane separators employed to minimize oxygen and substrate transport in MFCs generate a pH difference between the cathode and anode chambers. Depending on the type of membrane separator, there will be varying degrees of pH splitting. When CEM is used as a separator, MFCs experience more pH splitting than when AEM is used,^36,51^ as cations competing with protons for attachment to negatively charged functional groups in the anolyte when present in high concentrations. Additionally, anodic chamber protons build up as a result of anaerobes' biocatalytic activity, lowering the anolyte's pH.^41^ The acidic conditions present at anolytes inhibits the oxidation ability of bacteria, which further lowers proton generation.^32,83,84^ Due to the AEM surface's limited capacity for cation attachment, proton transfer rate is uncontrolled, which results in a decreased rate of pH splitting.

The rate-limiting step favors alkaline conditions because of its decrease in polarization changing the ideal pH range for the anode is from 7 to 9. Anaerobic bacteria activity is at its peak at neutral conditions, where the anode polarization resistance is the lowest.^29,63^ In order to keep the ideal pH of the anode solution, which is neutral for dual chamber MFC and slightly alkaline for air cathode MFC, and to maximize the anaerobic bacteria's catalytic activity, the appropriate buffer solutions must be used.^29,63,83,84^

Addressing issues like resistance, oxygen leakage, substrate migration, and pH imbalances is vital to unlocking the full potential of microbial fuel cells. By adopting improved membrane materials and optimizing system operations, these challenges can be mitigated, enabling better ion transport and microbial efficiency. Such advancements will not only boost the energy output and stability of MFCs but also enhance their applicability in sustainable energy production and environmental solutions. Continued research and innovation are essential for making MFCs a practical and efficient green technology.

## 8. Modification of membranes

Several research had previously shed light on how to enhance MFC performance by changing technical and engineering components.^85,86^ Despite impressive advancements in power generation, cathode catalysts, inconsistent system performance, limited electron recovery, the cost of the membranes continues to challenge the practical implementations. The shortcomings of highly desired membranes like Nafion include their expensive cost, minimal proton transport, and higher internal resistance as a result of easy fouling (due to their hydrophobic nature), among other things.^87^ High power densities may be achieved by MFC operation without a membrane, although coulombic efficiency will suffer because of uncontrollable substrate crossing and oxygen diffusion.^88^ As alternatives to expensive ion exchange membranes, a variety of materials including J-cloth, nylon mesh, glass fibre, and ceramic membranes were tried, but none were found to be suitable for a variety of reasons including biodegradation, high internal resistance and low coulombic efficiency, and high cost.^63,89,90^

Variety of organic polymeric substances, including cellulose, polysulfone, polyacrylonitrile, polyvinylidene fluoride, polyether ether ketone, polyimide, *etc.*, have a property known as hydrophobicity whereby the surface atoms repel the water molecules and prevent them from adhering. They are suitable for membranes because to this characteristic. A sudden rise in entropy during operation encourages the solute particles to accumulate on the membrane surface, clogging the membrane pores (membrane fouling), and ultimately leading to decreased MFC performance. In fact, the researchers were required to modify the membrane surface in order to improve the MFC performance due to the lack of an ideal material with excellent mechanical, chemical, and thermal stabilities, acid–base tolerance, microbial erosion resistance, *etc.* These developments and modifications are discussed below.

### 8.1 Modification of cation exchange membranes

The primary method of making CEMs is sulfonation. In order to reduce costs and address the Nafions shortage, sulfonated polymer membranes with sulfonate groups, such as SPEEK membranes^20,91^ and BPSH membranes, have recently been used to replace Nafions in MFCs.^86^ The natural PEEK polymers, a relatively affordable material with great chemical, thermal, and mechanical stability, are sulfonated to create SPEEK membranes, which are credited with strong proton conductivity.^92,93^ Although the SPEEK membrane had a lower oxygen mass transfer coefficient (1.6105 cm s^−1^), it had a higher value of power density (607 ± 14 mW m^−2^).^32^

Biofouling is reported to be decreased by BPSH membranes, which are hydrophilic in nature and have high DS and proton conductivity. Although the high DS of the BPSH membrane results in membrane expansion, electrolyte crossover and the concentration of other cations rather than protons ultimately result in a lower power density. The open structure enhances proton transfer and produces 16W m^−3^. But the oxygen crossover is extremely undesired for the catalytic activity of anaerobic bacteria. According to studies, the power density of the Nafion 112/poly aniline composite membrane is 124.03 mW m^−2^.^17^

To create multifunctional composite membranes with a high potential, the properties of the various membranes naturally are altered by blending. For instance, when SPEEK, a polymer with high conductivity, is blended with poly ether sulphone (PES), a cheap, low-conducting polymer membrane, the resultant composite membrane (PES/SPEEK 5%) exhibits remarkable MFC performance with a power density of 170 mW m^−2^ and CE of 68–76% compared to Nafion membranes.^94^

Additionally, 15% Fe_3_O_4_ nanoparticle/PES membrane, an inorganic filler/polymer composite moiety, demonstrated a power density of 20 mW m^−2^ and an open circuit voltage of 56 mV that were deemed superior to Nafions 117 membrane, for which the same were 15.4 mW m^−2^ and 610 mV respectively.^3^ Nafion 112 and Nafion 117 membranes, which are not part of the composite, had very low power densities.^26^ However, introducing polymer or nanoparticles into polymeric membranes may be damaging since intrusion may lead to distortion of the membrane's natural structure, particularly the surface roughness, which is reported to rise with increasing nanoparticle composition. PES-20% Fe_3_O_4_ membranes have rougher surfaces than PES-5% Fe_3_O_4_ membranes, which is likely caused by the large concentration of nanoparticles in them.^26^ However, a significant degree of surface roughness encourages membrane biofouling, which severely reduces MFC performance.^26^ Due to its affordability and higher power density compared to Nafions membrane, composite and nano-composite membrane production has attracted a lot of attention in recent years.^2,26,53^

Venkatesan and Dharmalingam (2015a)^10^ have reported on the MFC single-chamber operation using SPEEK sulfonated (polyether ether ketone) polymer. There have also been many composites of the same polymer that contain zeolite, iron oxide, and rutile titanium nanoparticles that have been researched and published (Table 1). This study compared its performance to that of conventional Nafion membrane. IER-based composite membranes had the highest conductivity of all the fillers tested.^17^

**Table 1:** List of CEMs and their modifications for MFC

Membrane	Thickness (mm)	Water uptake (%)	Ion exchange capacity (meq g^−1^)	Conductivity (S cm^−1^)	Oxygen permeability (cm s^−1^)	Power density	Chemical significance	Reference
SPEEK and Nafion 117	200	15.87	1.87	0.148 10^−3^	2.4 × 10^−6^	5.7 W m^−3^	Sulfonic acid groups (–SO_3_H) facilitate proton transport, enhancing ionic conductivity	86
SPEEK/sulfonated TiO_2_	120	39	1.05	1.382 × 10^−2^	0.8 × 10^−6^	1202 mW m^−2^	Sulfonation (–SO_3_H) enhances cationic transport and hydrophilicity	20
Sulfonated PSEBS/sulfonated SiO_2_	120	40	3.015	3.21 × 10^−2^	0.75 × 10^−5^	1209 mW m^−2^	(–SO_3_H) enhances cationic transport, hydrophilicity, and proton conductivity while improving membrane stability	95
SPSEBS/sulfonated TiO_2_	180	220	3.35	3.574 × 10^−2^	0.64 × 10^−5^	1345 mW m^−2^	Sulfonation (–SO_3_H) enhances cationic transport, hydrophilicity, and proton conductivity while sulfonated TiO_2_ improves mechanical strength and thermal stability	1
SPEEK/zeolite	180	15.83	1.47	0.148 × 10^−2^	4 × 10^−6^	176 mW m^−2^	Sulfonation (–SO_3_H) in SPEEK enhances proton conductivity and hydrophilicity, while zeolite improves ion selectivity and mechanical stability	10
SPEEK/rutile TiO_2_	180	21.83	1.98	0.167 × 10^−2^	2.2 × 10^−6^	98.1 mW m^−2^	Sulfonation (-SO_3_H) in SPEEK enhances proton conductivity and hydrophilicity, while rutile TiO_2_ improves mechanical strength, thermal stability, and oxidative resistance	10
SPEEK/Fe_3_O_4_	180	20.63	1.88	0.165 × 10^−2^	2.182 × 10^−8^	104 mW m^−2^	Sulfonation (-SO_3_H) in SPEEK enhances proton conductivity and hydrophilicity, while Fe_3_O_4_ improves magnetic responsiveness and mechanical stability	92
SPEEK/MMT	—	—	—	—	—	104 mW m^−2^	Sulfonation (-SO_3_H) in SPEEK enhances proton conductivity and hydrophilicity, while MMT (montmorillonite) improves mechanical strength, thermal stability, and barrier properties	29
Chitosan/MWCNT	240	0.7%	—	—	2.66 × 10^−4^	46.94 mW m^−2^	Chitosan enhances biocompatibility and film-forming ability, while MWCNT (multi-walled carbon nanotubes) improves electrical conductivity, mechanical strength, and thermal stability	93
SPEEK/PES	150	—	—	—	12.96 × 10^−4^	140 mW m^−2^	Sulfonation (-SO_3_H) in SPEEK enhances proton conductivity and hydrophilicity, while PES (polyethersulfone) improves mechanical strength, thermal stability, and chemical resistance	96
PVA/STA/GO	112	—	—	3.5 × 10^−2^	6.1 × 10^−6^	1.9 W m^−3^	PVA (polyvinyl alcohol) provides flexibility and film-forming ability, STA (silicotungstic acid) enhances proton conductivity, and GO (graphene oxide) improves mechanical strength, thermal stability, and hydrophilicity	97
SPEEK/STA	190	21.28	1.98	0.154 × 10^−2^	—	207 mW m^−2^	Sulfonation (-SO_3_H) in SPEEK enhances proton conductivity and hydrophilicity, while STA (silicotungstic acid) further boosts proton conductivity and oxidative stability	98
Zero gap CEM/MFC	183	—	1.85	—	—	3.1 W m^−2^	Zero-gap configuration in CEM enhances ion transport efficiency, reduces internal resistance, and improves overall MFC performance by minimizing energy losses	99
rGO/PVDF/CA	120	66	1.2	0.4	—	118 mW m^−2^	rGO (reduced graphene oxide) enhances electrical conductivity and mechanical strength, PVDF (polyvinylidene fluoride) provides chemical stability and hydrophobicity, while cellulose acetate improves flexibility and water retention	100
Zr MOF/PVDF (zirconium-based metal–organic framework)	—	—	2.41	—	—	13.8 mW m^−2^	Zr MOF enhances ion selectivity and proton conductivity, while PVDF (polyvinylidene fluoride) provides chemical stability and mechanical strength	39

**Fig. 5:**
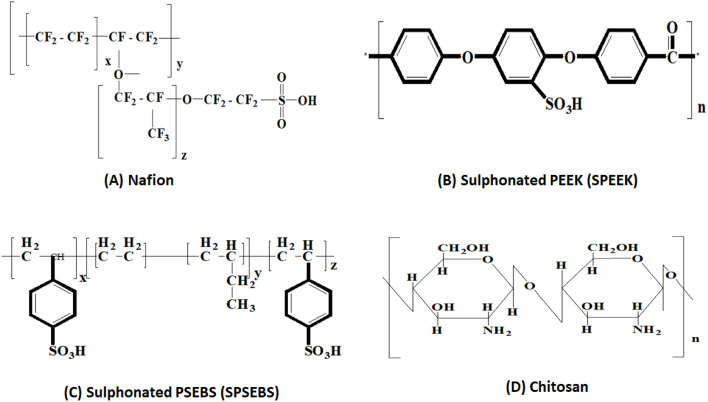
Structures of selected cation exchange membrane components of (A) nafion, (B) sulphonated PEEK (SPEEK), (C) sulphonated PSEBS (SPSEBS), and (D) chitosan.

The chemical significance of the membranes listed in Table 1 lies primarily in the incorporation of functional groups and fillers that directly influence ion transport, membrane stability, and overall MFC performance and the key cationic chemical structures are shown in Fig. 5. Sulfonation, through the introduction of sulfonic acid groups (–SO_3_H), significantly enhances proton conductivity and hydrophilicity, forming efficient ionic pathways for proton exchange. The integration of inorganic fillers such as TiO_2_, SiO_2_, Fe_3_O_4_, and Zr-based MOFs further contributes to improved thermal and mechanical stability, reduced oxygen permeability, and enhanced membrane durability under bioelectrochemical conditions. Nanomaterials like graphene oxide (GO) and MWCNTs increase electrical conductivity, provide structural reinforcement, and improve water uptake. Additionally, heteropolyacids like silicotungstic acid (STA) and microporous materials like zeolites enhance ion selectivity and oxidative resistance. These chemical modifications synergistically tailor membrane properties to meet the critical demands of microbial fuel cells, including long-term stability, ion exchange efficiency, and operational robustness.

Mokhtarian *et al.*, (2011)^101^ examined the Nafion 112/PANI membranes' composition in MFC. To make the composite, different depths of Nafion 112 were immersed in an aniline solution. The highest power density achieved was nine times more than the clean membrane and comparable to that of Nafion 117, a material that is routinely used. Yolcan *et al.*, 2023 (ref. 102) tested the PVDF/Nafion composite with various weights of electrospun PVDF and Nafion and observed a correlation. According to reports, 0.4 g of PVDF/Nafion membrane generated a maximum power density and coulombic efficiency that were even higher than those of Nafion 117. By Kim *et al.*, 2007 (ref. 17) using a phase inversion procedure, SPEEK was produced and composited with PES, a reasonably priced, low-conductivity polymer. The hydrophilic SPEEK increased the conductivity of the PES membrane. A PES/SPEEK of 5% demonstrated the maximum power density in MFC operation. A pore-filled PEM was described by Xi *et al.*, 2009 (ref. 103) employing etched polycarbonate.

### 8.2 Modification of anion exchange membranes

**Fig. 6:**
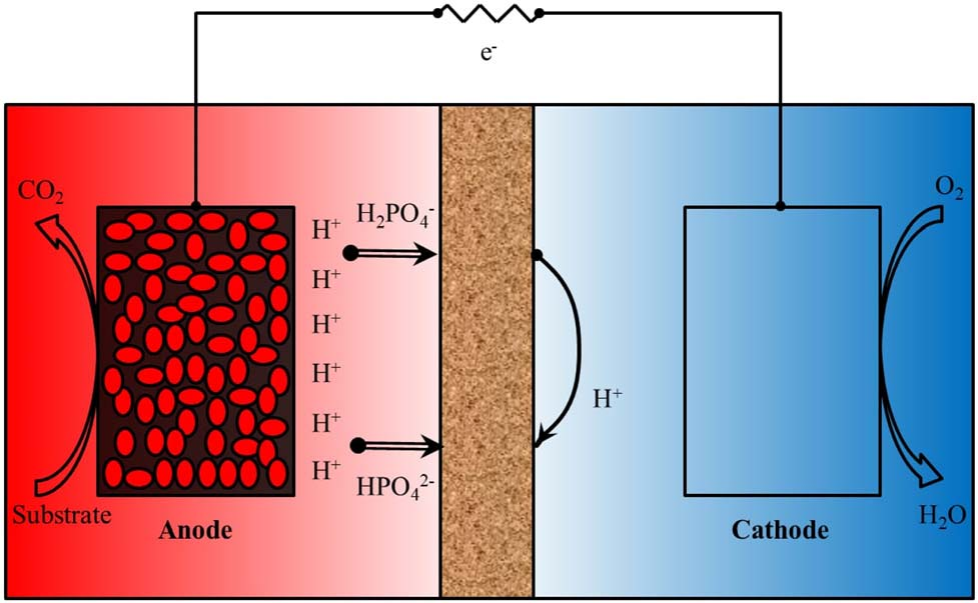
Mechanism of proton transfer *via* phosphate and bicarbonate.

AEMs consisting of polymer networks with immobilized, positively charged groups only let specific anions to pass from the cathode to the anode chamber, which is depicted in Fig. 6. For MFCs run with AEM, OH^−^ transport from the cathode to the anode achieves normal electro neutrality. However, it has recently been discovered that AEM significantly improves the performance through the action of chemical buffers.^32,50,57^ The phosphate buffer may combine with the protons created by microbial oxidation of organic materials at the anode chamber to create monobasic phosphate.^96^ AEM makes it simple to move monobasic phosphate from the anode to the cathode. As a phosphate buffer, monobasic phosphate is finally returned to the cathode.^96^

For mechanical stability, poly vinyl chloride (PVC) was typically used to strengthen the majority of commercially produced AEMs.^33^ Due to the dehydrochlorination of PVC, the prolonged use of such membranes has an impact on their physicochemical characteristics, ion exchange capacities, and permeability. These gradually turning black membranes inflated from increased water absorption and ripped off of the PVC fabric. When exposed to 1 M NaOH for even a short time, several AEMs, such AMX and AM1, change colour from light yellow to black.^91^ Due to the aforementioned technological difficulties, only a few number of AEMs have been successfully created and evaluated as AEMs for MFCs up until this point.^73^

AEM only outperformed all other membranes with a power density of 610 mW m^−2^ and a CE of 72%, which could be attributed to improved proton transport induced by phosphate anions and reduced internal resistance. According to the findings, AEM preserved charge balance by redistributing phosphate ions across the membrane, while orthophosphate anion species (HPO_4_^2−^ and H_2_PO_4_^−^) buffered the pH drop in the anode chamber. Rozendal *et al.* (2007)^12^ used AEM in an MFC and discovered that it reduced the pH gradient between the two chambers better than CEM (CEM pH = 6.4; AEM pH = 4.4). As cations (Na^+^, K^+^, Ca_2_^+^, Mg_2_^+^, and NH_4_^+^) travel through the membrane of CEMs and precipitate on the cathode surface, the current is typically reduced. In contrast, AEM-BES almost eliminates the likelihood of cation precipitation on the cathode surface while still allowing anions to pass from the cathode to the anode to maintain charge neutrality. According to Mo *et al.* (2009),^69^ utilising AEM in an MFC may result in stable power generation because the AEM successfully stops cation transport from the anodes, greatly reducing membrane resistance. In an MFC that employed wastewater, Rozendal *et al.* (2007)^12^ used AEM (Fumasep® FAB, FuMA-Tech GmbH) and discovered that AEM were superior to CEM like Nafion 117 in eliminating the pH gradient between two chambers.

Kim *et al.*, (2012)^41^ assessed the performance of an AEM (AMI-7001) in a dual chamber MFC and contrasted it with Nafion 117, CEM (CMI-7000), and Ultrafiltration membrane (UFM) with various molecular weight cut offs. AEM performed the best among the membranes with a PD 610 mW m^−2^ and CE of 72%. The movement of phosphate ions across the membrane appears to have helped AEM maintain a charge balance. According to Rozendal *et al.* (2007)^12^ AEM maintains the pH gradient between the two chambers due the free anionic migration. AEM in an MFC may aid stable power generation with lower membrane resistance due to less cation precipitation, according to Mo *et al.* (2009).^69^ Despite the fact that AEM reduces pH splitting, uncontrolled substrate crossover is still a drawback.^78^ This could promote the growth of biofilm on the cathode surface, which would reduce performance. The development of biofilm on the cathode surface could limit performance. Ion exchange between the electrodes is prevented by the biofilm, which continues to act as a physical barrier between the two chambers of the MFC. As a result, performance suffers due to an increase in the pH gradient. The fouled membranes must be replaced with new membranes for optimal performance, increasing operational costs.^8^

Several literature reported the MFC single-chamber operation employing various AEMs based on QPEEK (Mahendiravarman and Sangeetha, 2013), QPEI^83^ and QPSU^45^ with suitable ionic conductivity, sufficient mechanical, and chemical stabilities for MFC application. Such membranes were also modified to enhance their anti-biofouling activity using the appropriate modifiers modifiers (PDA modified QPEEK,^82^ AEOH modified QPEI^83^ and PDDA-functionalized GO-modified QPSU.^84^ The following findings were made regarding all three planned systems from the current analysis based on material attributes and performance data. In comparison to other combinations, QPSU/FGO-1.0% showed good performance with better power density, current density, anti-adhesive property, and anti-bacterial property. Out of the two anti-biofouling strategies used, it was discovered that the anti-adhesive strategy prevented bacteria from adhering to a membrane, while the anti-bacterial strategy killed bacteria that were already on the membrane (Table 2).

**Table 2:** List of AEMs and their modifications for MFC

Membrane	Thickness (μm)	Water uptake (%)	Ion exchange capacity (meq g^−1^)	Conductivity (mS cm^−1^)	Oxygen permeability (cm s^−1^)	Power density	Chemical significance	Reference
AFN (commercially available membrane)	150	—	2.0–3.5	—	1.26 × 10^−4^	0.38 mA cm^−2^	Offers high ionic conductivity, chemical stability, and selective anion transport, making it suitable for electrochemical applications like MFCs	87
QA-AEM (quaternary ammonium anion exchange membrane)	—	92.8 ± 2.8	2.46	—	—	16 mW m^−2^	It facilitates selective anion transport, enhances ionic conductivity, and provides chemical stability for efficient electrochemical performance in MFCs	1
RALEX AEM	450	—	—	—	—	57.8 mW m^−2^	It ensures effective anion transport, high chemical resistance, and durability, making it suitable for long-term MFC applications	1
PVA-PDDA (polyvinyl alcohol-poly(diallyldimethylammonium chloride))	110	1.08	0.83	2.2 × 10^−2^	7.57 × 10^−8^	6.68 W m^−3^	Enhances ionic conductivity, provides mechanical stability, and improves anion exchange capacity for efficient MFC performance	90
QPEEK/PDA (quaternized polyether ether ketone/polydopamine)	30	22	1.39	12	2.1 × 10^−5^	918 mW m^−2^	Enhances anion exchange capacity, improves hydrophilicity, and provides better chemical stability for efficient ion transport in MFCs	84
QPEI/AEOH (quaternized polyethyleneimine/ammonium ethanol hydroxide)	30	42 ± 3	0.968	2.4 × 10^−3^	2.3 × 10^−5^	620 mW m^−2^	Enhances anion exchange capacity, improves ionic conductivity, and provides better chemical stability for MFC applications	83
QPSU/GO (quaternized polysulfone/graphene oxide)	30	43.34	1.45	1.80 × 10^−2^	2.1 × 10^−5^	1036 mW m^−2^	Enhances anion exchange capacity, improves mechanical strength, and increases ionic conductivity for efficient MFC performance	84
QPVA/TiO_2_ (quaternized polyvinyl alcohol/titanium dioxide)	43	—	—	1.4 × 10^−2^	2.3 × 10^−5^	125.4 mW m^−2^	Enhances hydroxide ion conductivity, provides antibacterial properties, and improves chemical stability for efficient MFC performance	13
Zero gap AEM/MFC	106	—	1.85	—	—	8.8 W m^−2^	Enhances proton transfer efficiency, reduces internal resistance, and improves power density by minimizing the electrode-membrane distance in microbial fuel cells	99
AEM/MFC (anion exchange membrane/microbial fuel cell)	—	—	—	—	—	0.11 W m^−2^	It facilitates selective anion transport, reduces pH imbalance, and enhances ion exchange efficiency for improved MFC performance	104
PVA/AEM/MFC (polyvinyl alcohol/anion exchange membrane/microbial fuel cell)	—	2.21 g g^−1^	0.91	2.4 × 10^−2^	—	—	It enhances proton conductivity, provides mechanical stability, and improves ion selectivity for efficient MFC operation	42

**Fig. 7:**
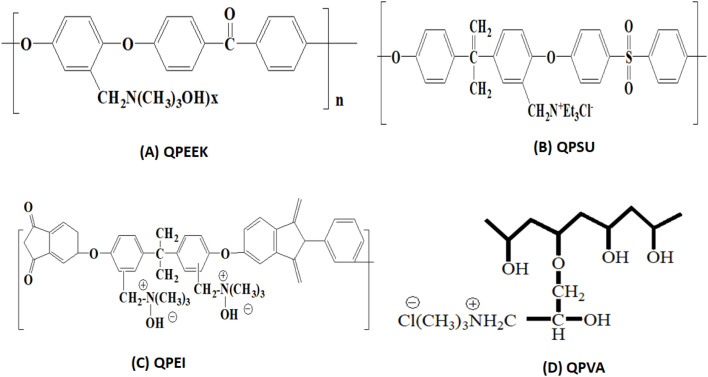
Structures of selected anion exchange membrane components of (A) QPEEK, (B) QPSU, (C) QPEI, and (D) QPVA.

The chemical significance of the anion exchange membranes (AEMs) listed in Table 2 lies in their functional modifications, which are designed to enhance ion transport, chemical stability, and membrane durability in microbial fuel cell (MFC) environments the major anionic chemical structures are shown in Fig. 7. AEMs such as AFN, RALEX, and QA-AEM utilize quaternary ammonium groups (–NR_4_^+^) to facilitate selective hydroxide ion (OH^−^) transport, ensuring effective charge balance across the membrane while maintaining alkaline stability. Polymers like QPEEK, QPSU, and QPEI, when quaternized, exhibit significantly improved anion exchange capacity and enhanced hydrophilicity, which promote ionic mobility and water uptake, critical for sustained electrochemical reactions. The integration of graphene oxide (GO) in QPSU or TiO_2_ in QPVA further augments mechanical strength, antibacterial properties, and oxidative stability. Moreover, blending polyvinyl alcohol (PVA) with polyelectrolytes like PDDA or using dopamine-based adhesion layers (*e.g.*, QPEEK/PDA) improves membrane flexibility and surface functionality, aiding biofouling resistance. These chemical modifications ensure robust ionic pathways, high conductivity, and long-term operability, all essential for efficient and durable MFC performance.

## 9. Comparative analysis

Many researchers prefer polymeric membranes for microbial fuel cells because they have various advantages over alternative varieties. One significant benefit is their affordability; compared to ceramic or composite membranes, polymeric membranes are frequently less expensive to produce, allowing for a wider use in MFC applications. Their adaptable architecture makes it simple to adjust to particular MFC specifications, like ion selectivity, conductivity, and durability, so maximizing overall performance. Additionally, polymeric membranes are readily modifiable to improve performance, for example, by altering the surface to increase selectivity or decrease fouling. Furthermore, a lot of polymeric membranes have strong ionic conductivity, which is essential for effective ion transport between MFC chambers. Long-term stability and performance are guaranteed by their mechanical robustness, even under challenging MFC circumstances. Polymeric membranes are also an environmentally friendly and scalable option for MFCs due to its scalability in industrial settings.^43,44^

Studies on MFC have been carried out with a different mode of reactors and experimental variables (*e.g.* MFC architecture, types of electrodes, substrate type, dominant ARB, *etc*). Understanding the precise effect of membrane functions on MFC performance is difficult because of this diverse methodology. The effectiveness of Nafion has traditionally been utilised as the standard for evaluating the viability of subsequently developed membranes. The parameters used to assess membranes technically include proton exchange capacity and oxygen permeability, substrate loss, electrode energy losses, and cathode kinetics.

In comparison to a few PEMs and AEMs (UltrexTM CMI-7000, Selemion, and SPEEK), high molecular cut-off weight membranes such as MFM, Nafion, and UFM displayed much higher oxygen permeability during MFC operation. Substrate loss is higher in MFM and UFM than in the majority of ion exchange membranes, presumably due to the membrane's inclusion of relatively large pores (IEMs). Nafion membrane might not be the optimal solution for MFC applications due to its high cost. The excellent performances of MFCs with dense AEMs, including their high-power density outputs, high coulombic efficiencies, and exceptional stability, have been reported in numerous investigations. The following reasons are that the reduced oxygen crossover and low gas permeability of AEMs, especially when compared to Nafion (CEMs), help maintain anaerobic conditions at the anode, essential for sustained high current efficiencies (CEs). Additionally, AEM-based systems minimize “pH splitting,” an unfavorable occurrence that causes a significant pH gradient across the membranes, thus preventing large pH changes. The relatively low resistances at the cathode further enhance the system's efficiency. Moreover, the precipitation of reduced compounds from cations is avoided, and due to the low internal ohmic resistances of AEMs compared to CEMs, these membranes exhibit high ionic conductivities, further improving performance. However, high substrate crossover is undoubtedly a drawback for AEMs. This could promote the growth of biofouling on cathode surfaces, lowering performance. There is currently no perfect AEM for MFCs, so we must choose ones built specifically for our needs.

## 10. Future perspectives

The membrane functions as an ionic conductor, ion transporter, gas and electron barrier, and plays a crucial part in the functionality and efficiency of MFCs. The mechanical stability and ionic conductivity of the membrane are important factors that determine MFC performance. The difficulty in creating efficient AEMs lies in achieving high ionic conductivity while retaining mechanical stability. The membrane's chemical stability is directly impacted by the type of cationic group selected. Furthermore, scalability and material costs have a practical role in the problem of creating thinner membranes without sacrificing other properties. In general, solving these issues will be critical to improving the creation of customized polymeric membranes for MFCs, which will allow for more effective and long-lasting energy conversion. Exciting opportunities exist for improving performance, efficiency, and useful uses of customized polymeric membranes in MFCs in the future.

Research in MFCs is progressing towards improving membrane ion transport properties, with a particular focus on enhancing proton conductivity and selectivity to increase power output and efficiency. This involves exploring novel materials and membrane architectures designed to optimize these properties. Similarly, biofouling presents a challenge by hindering membrane performance, as biofilm buildup can reduce efficiency. To counter this, researchers are investigating antifouling membranes and surface modification techniques to prevent biofilm formation and improve long-term reliability and efficiency. Future developments in MFC technology may concentrate on improving membrane durability to withstand harsh operational conditions, ensuring system longevity. Innovations in membrane composition and design will enhance the lifespan and performance of MFCs. Additionally, cost-effective materials and manufacturing processes will make MFC technology more affordable, driving its adoption for renewable energy generation and wastewater treatment.

Another key area of research is scaling up MFC systems and integrating them into existing infrastructure, particularly in large-scale wastewater treatment applications. Advances in system optimization and membrane design will facilitate the deployment of MFCs in practical settings. Furthermore, ongoing research is exploring tailored membranes designed to meet the specific needs of various MFC configurations. The integration of emerging technologies, such as nanotechnology and additive manufacturing, will likely lead to the development of advanced materials and structures, enhancing MFC performance and making them more viable for large-scale sustainable energy production and wastewater management.

All things considered, the future of customized polymeric membranes in MFCs appears bright, with continuous research and development propelling improvements in membrane architecture, functionality, and uses. In order for MFCs to fulfill their full potential as effective and sustainable energy conversion devices, these membranes will be essential.

## 11. Conclusion

The overall performance of an MFC system is significantly influenced by the characteristics of the membrane, a key component that dictates ion transport, gas separation, and system stability. While several factors, including system architecture, electrode material, bacterial species, organic matter composition, and operational parameters (*e.g.*, pH, conductivity, and catholyte type), contribute to efficiency, the applicability of a membrane is ultimately determined by its performance, durability, and cost-effectiveness. A high-performing membrane must effectively balance ionic conductivity, mechanical stability, and oxygen permeability to maintain optimal electron transfer and power output.

One of the primary challenges in developing AEMs is achieving high ionic conductivity (≥100 × 10^−3^ S cm^−1^) while ensuring sufficient mechanical strength and chemical stability. Although increasing charge density enhances conductivity, excessive water uptake can weaken the mechanical integrity of the membrane, necessitating a trade-off between these properties. Careful selection of cationic groups is crucial, as they directly impact the chemical stability and ionic mobility within the membrane. Additionally, the fabrication of thinner membranes without compromising oxygen permeability or scalability remains a significant challenge. Future advancements in material innovation and membrane design will play a critical role in overcoming these limitations, paving the way for more efficient and cost-effective MFC systems.

## Abbreviations

AEM: Anion exchange membrane
BPM: Bipolar membrane
CO_2_: Carbon dioxide
CEM: Cation exchange membrane
*E*
: Cell potential
cm^−1^: Centimeter
COD: Chemical oxygen demand
CE: Coulombic efficiency
DO: Dissolved oxygen
BPSH: Disulfonated poly(arylene ether sulfone)
DCMFC: Dual chamber microbial fuel cell
e^−^: Electron
eV: Electron volt
AEOH: Ethanolamine
F: Faraday
FC: Fuel cell
g cm^−2^: gram per centimeter square
g L^−1^: gram per liter
GO: Graphene oxide
H: Hours
H_2_: Hydrogen
H^+^: Hydrogen ion or proton
OH^−^: Hydroxyl ion
IEC: Ion exchange capacity
IEM: Ion exchange membrane
IPA: Isopropyl alcohol
K: Kelvin
kg m^−3^: Kilogram per cubic meter
kg m^−3^: Kilogram per meter cube
kW: Kilowatt
LSV: Linear sweep voltammetry
KA: Mass transfer coefficient
MHz: Mega hertz
MPa: Mega pascal
MEA: Membrane electrode assembly
m^2^: Meter square
MFM: Micro filtration membrane
MEC: Microbial electrolysis cell
MFC: Microbial fuel cell
μm: Micrometer
Mm: Milli meter
mS cm^−1^: Milli Siemens per centimeter
mV s^−1^: Milli volt per second
mV: Milli volte
mW: Milli watt
mA m^−2^: Milliamps per square meter
mA cm^−2^: Milliamps per square centimeter
meq g^−1^: Milliequivalent per gram
mg L^−1^: Milligram per liter
mg cm^−2^: Milligram per square centimeter
mL: Milliliter
mL min^−1^: Milliliter per minute
Mmol: Millimol
mW m^−2^: Milliwatts per square meter
Min: Minute
M: Molar
MWCNT: Multiwalled carbon nanotube
NASA: National Aeronautics and Space Administration
NMP: *N*-Methyl pyrollidone
Ω: Ohm
*R*_s_
: Ohmic resistance
OCV: Open circuit voltage
O_2_: Oxygen
*D*_O_
: Oxygen diffusion coefficient
*K*_O_
: Oxygen mass transfer coefficient
ORR: Oxygen reduction reaction
ppm: Parts per million
%: Percentage
PBS: Phosphate buffer solution
Pt: Platinum
Pt/C: Platinum on carbon
PI: Ployimide
*R*_p_
: Polarization resistance
PDDA: Poly diallyl dimethyl ammonium chloride
PEEK: Poly ether ether ketone
PEI: Poly ether imide
PES: Poly ether sulphone
SPSU: Poly sulphone
PTFE: Poly tetra fluoro ethylene
PTFE: Poly tetra fluro ethylene
PVC: Poly vinyl chloride
PAN: Polyacrylonitrile
PDA: Polydopamine
PI: Polyimide
PSEBS: Polystyrene ethylene butylene polystyrene
PSU: Polysulphone
PVDF: polyvinylidene fluoride
KCl: Potassium chloride
KOH: Potassium hydroxide
PEM: Proton electrolyte membrane
PEMFC: Proton exchange membrane fuel cell
QPSU: Quaternized poly sulphone
Re (Z): Real part
RPM: Revolution per minute
SEM: Scanning electron microscopy
S cm^−1^: Siemen per centimeter
SiO_2_: Silicon dioxide
Ag/AgCl: Silver silver chloride
SCMFC: Single chambermicrobial fuel cell
NaCl: Sodium chloride
NaOH: Sodium hydroxide
cm^2^ s^−1^: Square centimeter per second
m2: Square meter
SnCl_4_: Stannic chloride
SPEEK: Sulfonated poly ether ether ketone
THF: Tetrahydrofuran
TGA: Thermogravimetric analysis
TiO_2_: Titanium dioxide
TEA: Triethyl amine
UFM: Ultra filtration membrane
UK: United Kingdom
V: Volts
H_2_O: Water
Wt: Weight
wt%: Weight percentage
XRD: X-ray diffraction
XPS: X-ray photoelectron spectroscopy
ZrO_2_: Zirconium oxide

## Data availability

No primary research results, software or code have been included and no new data were generated or analysed as part of this review.

## Conflicts of interest

There are no conflicts to declare.
